# Effectiveness of Different Sample Treatments for the Elemental Characterization of Bees and Beehive Products

**DOI:** 10.3390/molecules25184263

**Published:** 2020-09-17

**Authors:** Maria Luisa Astolfi, Marcelo Enrique Conti, Elisabetta Marconi, Lorenzo Massimi, Silvia Canepari

**Affiliations:** 1 Department of Chemistry, Sapienza University, Piazzale Aldo Moro 5, I-00185 Rome, Italy; l.massimi@uniroma1.it (L.M.); silvia.canepari@uniroma1.it (S.C.); 2 Department of Management, Sapienza University, Via del Castro Laurenziano 9, I-00161 Rome, Italy; marcelo.conti@uniroma1.it; 3 Department of Public Health and Infectious Diseases, Sapienza University, Piazzale Aldo Moro 5, I-00185 Rome, Italy; elisabetta.marconi@uniroma1.it

**Keywords:** sample preparation, trace element, toxic element, spectroanalytical technique, biomonitoring

## Abstract

Bee health and beehive products’ quality are compromised by complex interactions between multiple stressors, among which toxic elements play an important role. The aim of this study is to optimize and validate sensible and reliable analytical methods for biomonitoring studies and the quality control of beehive products. Four digestion procedures, including two systems (microwave oven and water bath) and different mixture reagents, were evaluated for the determination of the total content of 40 elements in bees and five beehive products (beeswax, honey, pollen, propolis and royal jelly) by using inductively coupled plasma mass and optical emission spectrometry. Method validation was performed by measuring a standard reference material and the recoveries for each selected matrix. The water bath-assisted digestion of bees and beehive products is proposed as a fast alternative to microwave-assisted digestion for all elements in biomonitoring studies. The present study highlights the possible drawbacks that may be encountered during the elemental analysis of these biological matrices and aims to be a valuable aid for the analytical chemist. Total elemental concentrations, determined in commercially available beehive products, are presented.

## 1. Introduction

Various natural and anthropogenic emission sources of toxic elements may cause air pollution [1,2,3,4,5,6]. Among different air pollution monitoring techniques, biomonitoring has recently become one of the most widely used technique, due to its ease of operation, low cost, efficiency and specificity [7,8,9,10]. In fact, several living organisms, known as biomonitors, can accumulate toxic elements, allowing the monitoring of pollutants concentrations in the environment for integrated measurements over time [11,12,13]. The use of apis mellifera and beehive products for biomonitoring studies has been widely investigated [14,15,16,17,18,19,20] and reviewed [21,22,23]. Honeybees and the associated matrices are often considered as efficient sentinels for environmental biomonitoring [7,17]. Trace elements can be transferred to honeybees and beehive products from all the environmental compartments (soil, vegetation, air and water) in the areas covered by forager honeybees [24], within which honeybees and beehive products may supply integrated representative samples [25]. Measurements of element concentrations in honey samples are relevant for the healthiness assessment of the honey in terms of the presence of essential metals and for ensuring the human health safety by assessing the admissible levels of toxic elements [21]. Moreover, the assessment of element concentrations in honey is also useful for its classification based on its genuineness and its botanical and geographical origins [21].

Among the different instrumental methods used for the determination of elements in honeybee and beehive products, atomic and mass spectrometry are considered as the most sensitive, accurate, and robust techniques, thus being routinely and customarily applied [21,26,27]. Flame atomic absorption spectrometry (F-AAS) has been often employed for the low-cost and rapid determination of high concentrations of metals [21,22,28]. To control the quality of honey and other beehive products in terms of contamination by heavy metals, many sensitive techniques are required, including graphite furnace atomic absorption spectrophotometry (GF-AAS) [17,19], electrothermal atomic absorption spectrometry (ET-AAS) [29,30], microwave plasma technique atomic emission spectrometry (MP-AES) [31], inductively coupled plasma optical emission spectrometry (ICP-OES) [14,16,24,32,33,34], and inductively coupled plasma mass spectrometry (ICP-MS) [14,18,20,35,36,37,38,39]. In addition, other atomic techniques, such as atomic analyzer mercury (AMA), hydride generation-atomic absorption spectrometry (HG-AAS) and cold vapor atomic fluorescence spectrometry (CV-AFS), have been employed in environmental studies for the determination of Hg, As and Se [40,41].

The analysis of biological samples by atomic and mass spectrometry techniques is a difficult and challenging task [21]. Honey and other beehive products are very complex organic matrices with problems related to the sample heterogeneity, selection of sample treatment, and decomposition as well chemical interferences during measurements [21,22,38]. Moreover, bees and beehive products are matrices with high C contents [42,43,44] and their incomplete sample decomposition may cause a residual carbon content (RCC) in the final digests. During ICP analysis, the element signals with a similar ionization potential to that of C is enhanced due to C charge transfer reactions [45,46]. The residual acidity in the final digests is also important for the polyatomic interference (for ICP-MS), nebulization efficiency and for reducing the instrument interface damage [47,48,49]. Samples of honey and other beehive products are commonly decomposed using high temperature dry ashing [17,50] or wet digestion procedures, in order to destroy the carbohydrate-rich sample matrix and minimize the matrix-based interferences [21,22]. To obtain an efficient honeybee and beehive products digestion, various reagents or mixtures are used, including concentrated HNO_3_ or other acids (such as HCl, HClO_4_ and H_2_SO_4_), frequently mixed with 30% H_2_O_2_, using open vessels or sealed quartz or polytetrafluoroethylene (PTFE) vessels in microwave-assisted systems [21,22,35,38,39]. Unfortunately, to date the validation of methods and procedures used for the analysis of bees and beehive products is difficult because no certified reference material (CRM) of these matrices is available. Instead, the trueness of the methods and procedures applied is commonly checked by the analysis of other CRMs containing high levels of carbohydrates, such as Antarctic krill (MURST-ISSA2), apple leaves (NIST 1515), brown bread (BCR 191), corn (NBS 8413), mixed Polish herbs (INCT-MPH-2), tea leaves (INCT-TL-1) and wheat (IPE 684) or whole meal flour (BCR 189) [21,22]. For the same purpose, the recovery tests are carried out on the chosen samples spiked with known amounts of selected elements [21,22,35].

Generally, among all beehive products, only honey [14,20,21,23,32,34,51,52,53] or honey and pollen [17,24,35] are considered in the optimization approach for elemental determination with ICP techniques. To our knowledge, in the literature, few studies are focused on the elemental content of other beehive products such as propolis [19,31,38] or royal jelly and beeswax [15].

Therefore, the aim of this study is to compare different methods of sample preparation, including two systems (microwave oven and water bath) and different mixture reagents, which will allow the assessment of the exposure of bees to different element concentrations and the quality control of beehive products in terms of contamination made by toxic elements in both routine and large-scale investigations. The optimized methods were employed to determine 40 elements (Al, As, B, Ba, Be, Bi, Ca, Cd, Co, Cr, Cs, Cu, Fe, K, Li, Mg, Mn, Mo, Na, Ni, P, Pb, Rb, Sb, Se, Si, Sn, Sr, Te, Ti, Tl, U, V, and Zn) with ICP techniques. The analytical performance and quality control of the optimized procedures were evaluated on CRMs and field samples of bees, beeswax, honey, honeydew, pollen, propolis and royal jelly. For this purpose, different commercially available beehive products were analyzed.

## 2. Results and Discussion

### 2.1. Preliminary Evaluation of Digestion Efficiency

Preliminary tests were carried out in order to evaluate the effects of the reagent mixture, temperature and pressure on sample digestion, which was evaluated in digests by the RCC and residual acidity determination. HNO_3_ and H_2_O_2_ (methods A and B) were preferred to other reagents (HCl, HClO_4_, or H_2_SO_4_) because they allow the oxidation of almost all organic compounds and cause minor spectral interferences or problems in ICP-MS [49]. The aqua regia digestion procedures (methods B1 and B2) were chosen for assessing the total recoverable elements in all samples [54,55,56]. The total elemental content is important information to evaluate and control the quality of honey and other beehive products in terms of contamination. A mixture of aqua regia has been widely used for the digestion of various solid wastes such as ashes, sludge, sediments and soils [54,56]. In addition, HF is effective in extracting silicate-bound elements [54,56]. To improve element recovery and to increase the reaction kinetics, oxidizing agents such as H_2_O_2_ were added in the digestion procedures [56,57]. The comparison of the results obtained by using the different sample digestion procedures (methods A, B, B1 and B2) allows for the evaluation of the elements that have not been completely recovered in the various considered matrices.

**Figure 1:**
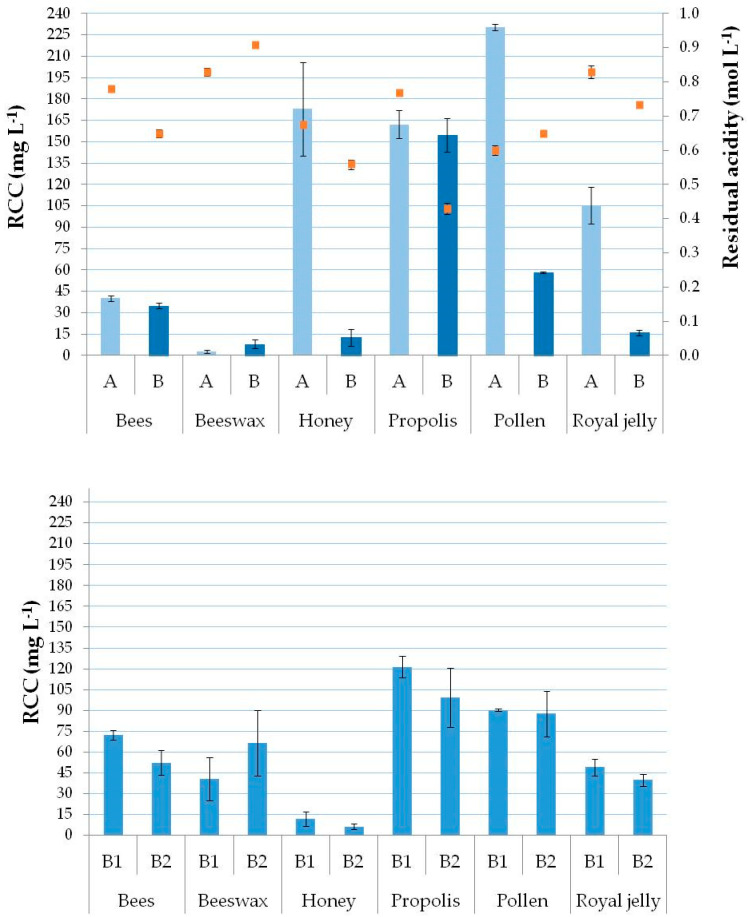
Sample treatment (A = open-vessel digestion heated with water bath, B = closed-vessel microwave-assisted digestion with HNO_3_/H_2_O_2_ mixture, B1 = closed-vessel microwave-assisted digestion with aqua regia/H_2_O_2_ mixture, and B2 = closed-vessel microwave-assisted digestion with aqua regia/HF mixture) effect on bees and beehive products digestion efficiency. Bars represent residual carbon content (RCC; left Y axis; *n* = 3) and line (-□-) represents residual acidity (right Y axis; *n* = 3).

A selected tolerance level of the RCC in solution, lower than 200 or 2000 mg L^−1^, was considered appropriate for the subsequent analyses by ICP-MS or ICP-OES [58,59]. Preliminary experiments were performed using a fixed amount of 200 mg of the sample to evaluate the minimum HNO_3_ amount that was sufficient to obtain suitable values of the RCC in the digests with methods A and B. In methods A and B, 67% HNO_3_ was varied in order to achieve an efficient organic matter digestion, using an acid solution with a concentration as low as possible. Thus, digestion using two different 67% HNO_3_ amounts (1 or 2 mL) was tested. Using 1 mL HNO_3_, final digests presented a yellow color with solid residues remaining as suspended particles. All final digests obtained using 2 mL HNO_3_ presented a colorless aspect, except for beeswax, which presented solid residues for both methods A and B. Thus, the RCC in digests (Figure 1) was lower than 231 ± 2 mg L^−1^ in the pollen digests by method A, and 155 ± 12 mg L^−1^ in the propolis digests by method B. The digestion efficiency was high when using both methods B1 and B2 with an RCC lower than 121 ± 8 and 99 ± 21 mg L^−1^ (in the propolis digest), respectively.

The residual acidity was also determined in the final digests obtained from the digestion procedure performed with the HNO_3_/H_2_O_2_ mixture. The residual acidity was in the range 0.429 ± 0.016–0.909 ± 0.007 mol L^−1^ using microwave-assisted digestion, which showed a good digestion efficiency, while the activity was in the range 0.599 ± 0.016–0.829 ± 0.018 mol L^−1^ using water bath-assisted digestion. The results suggested that all the digests obtained using the four treatments were suitable for ICP-OES and ICP-MS analyses, except for pollen digests obtained by method A for ICP-MS analysis. Comparing the element concentrations in the digests obtained with all four digestion treatments, it is possible to verify if there is polyatomic ion interference due to the C concentrations in the digested pollen by method A and analyzed with ICP-MS.

### 2.2. Selection of ICP Instrument

ICP-MS analysis is generally more susceptible to interferences than ICP-OES. Spectral interference in ICP-MS may be polyatomic and isobaric due to the presence of nonanalyte elements of a similar mass, such as species produced by plasma gas (Ar), atmosphere, nebulizer gas and matrix or combinations thereof [58,60]. Elemental analyses of biological matrices are commonly subjected to interferences caused by major constituents such as C, Ca, Cl, Mg, N, Na, and S [61,62]. The occurrence of spectral effects during the complex sample analysis seriously interferes with the determination of many isotopes, mainly up to 100 amu [60,61,62,63,64]. Therefore, the collision-reaction interface (CRI) mode was used for the determination of As, Ca, Co, Cr, Fe, Mn, Ni, S, and Se in bees and beehive product samples, using H_2_ and He as cell gases. The first high ionization potential of S (10.357 eV) leads to a relatively low ionization efficiency in an Ar-based plasma [65]. The 34S isotope was selected for the determination of total S because of its lower spectral interferences [65]. The main drawback of the use of the CRI is the reduction in sensitivity when the collision or reaction gases are employed [65]. The best gas flow rates selected for S were 90 mL min^−1^ H_2_ and 0 mL min^−1^ He to the skimmer and sampler cone, respectively. For this element, a comparison with ICP-OES was made. As shown in Figure S1, the correlation between the S data obtained with ICP-MS and ICP-OES is not good. Hence, the ICP-OES determination of S was preferable because it not affected by spectral interferences. The best compromise was obtained for As, Ca, Cr, Fe, Mn, and Se with a mixture of 30 mL min^−1^ He and 70 mL min^−1^ H_2_ to the sampler and skimmer cones, respectively, in agreement with previously reported methods [60]. There were few differences between the standard and CRI mode measurements of 59Co and 60Ni; therefore, these elements were analyzed in the standard mode.

The wide differences in the potential interferences and concentration ranges of the elements revealed that the use of both ICP techniques increases the accuracy for some elements. Specifically, the As, Be, Bi, Ce, Ga, Li, Nb, Pb, Sb, Se, Sn, Te, Tl, U, W, and Zr content of most samples were below the limit of detection (LOD) for the ICP-OES analysis (Tables S1–S6); whereas very high concentrations of some macroelements, especially K, caused signal saturation in the ICP-MS analysis and required at least one additional dilution level for the analysis. Elements in the digests obtained by methods A and B were analyzed by both ICP-OES and ICP-MS instruments, while elements in the digests obtained by methods B1 and B2 were analyzed by ICP-OES only, to avoid the effect of interference in ICP-MS due to the presence of HCl or HF.

### 2.3. Analytical Performances

#### 2.3.1. Linearity

The linearity ranges for ICP-MS and ICP-OES analyses are shown in Table 1 and Table S7, respectively. The obtained correlation coefficients of all calibration curves were >0.99 and a good linearity was confirmed by a Mandel test [66].

#### 2.3.2. Limit of Detection and Quantification

The LOD and limit of quantification (LOQ) for each element are shown in Table 1 and Table S7. LOD and LOQ values were of a similar magnitude to those previously reported [67,68]. With Regulation No. 2015/1005 [69], the European Union fixed a maximum level of 0.10 mg kg^−1^ for Pb in honey. No regulated standards are available to evaluate the other element levels in honey samples; therefore, Codex Alimentarius [70] stated that “honey shall be free from heavy metals in amounts which may represent a hazard to human health”. Consequently, there is the need to determine very low concentrations of elements that may be present in honey in trace and ultratrace levels. The tested analytical methods using ICP-MS were sufficiently sensitive to quantify all the selected elements in bees and beehive products, including Pb that has an LOD value (0.001 mg kg^−1^) 100 times lower than the maximum accepted level for honey [69].

#### 2.3.3. Precision, Trueness and Recovery Study

An apple leaf CRM was used to evaluate the trueness and precision under the repeatability of the tested methods (Tables S8 and S9). This CRM was used because there are no suitable reference materials for bees and beehive products and because it is frequently used [21]. However, the matrix of the apple leaf CRM is a powder and has a chemical and elemental composition different from the bees and beehive products. According to Pohl et al. (2009) [21], the use of reference materials that contain large amounts of C or carbohydrates can be very useful for the validation of the honey analysis method. Certified values of As, Be, Bi, Cs, Ga, Li, Nb, Se, Si, Sn, Te, Ti, Tl, and Zr concentrations were not available for the CRM used so further testing is required for validation. As shown in Table S9, the trueness bias percentage and repeatability obtained by method B1 were estimated in the range from −9.7 (La) to 12.6% (Co) and from 2.6 (S) to 25% (Sb), respectively, and were improved from those obtained by the other tested methods (A, B and B2). The results of As, Cr and Se in the digests obtained by method B1 and analyzed by ICP-MS are higher because they are strongly affected by the interference due to Cl. For this reason, we have chosen to report the data obtained with ICP-OES. To compare the observed results with the certified concentration of the CRM, the Z-scores were calculated [71]. Table S8 shows that the Z-scores of all elements excluding Cd, Rb and S obtained by method B1 were smaller than 2, thus the results are considered acceptable. For Cd and Rb, the results obtained by method B1 were underestimated compared to the certified values (Z-score < −2), whereas they rather tend to be overestimated for S.

With the lack of a suitable CRM, spiked samples were also used to determine the elemental recoveries by methods A and B, in accordance with previous studies [35]. It is worth mentioning that the study of recoveries does not allow for the assessment of the efficiency of the digestion procedure in decomposing the sample matrix but only enables the evaluation of matrix effects and losses or increases in a concentration of elements compared to the added amounts. All six sample matrices were spiked with two concentrations (the third and fifth instrumental calibration standards) before sample digestion. Recoveries for all elements fell within 20% of the expected value, with many of the elements recovering within 10%, excluding Al, Ca, Ce and Zn in beeswax, Ce in royal jelly, Ba, Cs, Ga, K, Mn, Na, Nb, P, Ti and Zn in bees, and Al, As, Si, Ti and Tl in propolis by both methods A and B, which fell within 30% (Table 2). The within-run precision for all the elements in honey and royal jelly and for most of the elements in other matrices was less than 10%. The intermediate precision was less than 15% for most of the elements in all matrices excluding As and Se in the digests by method A, and As, Ba, Be, Bi, Cd, Cr, Pb and Te in the digests by method B (Table 2).

#### 2.3.4. Mixture Reagent Digestion

**Figure 2:**
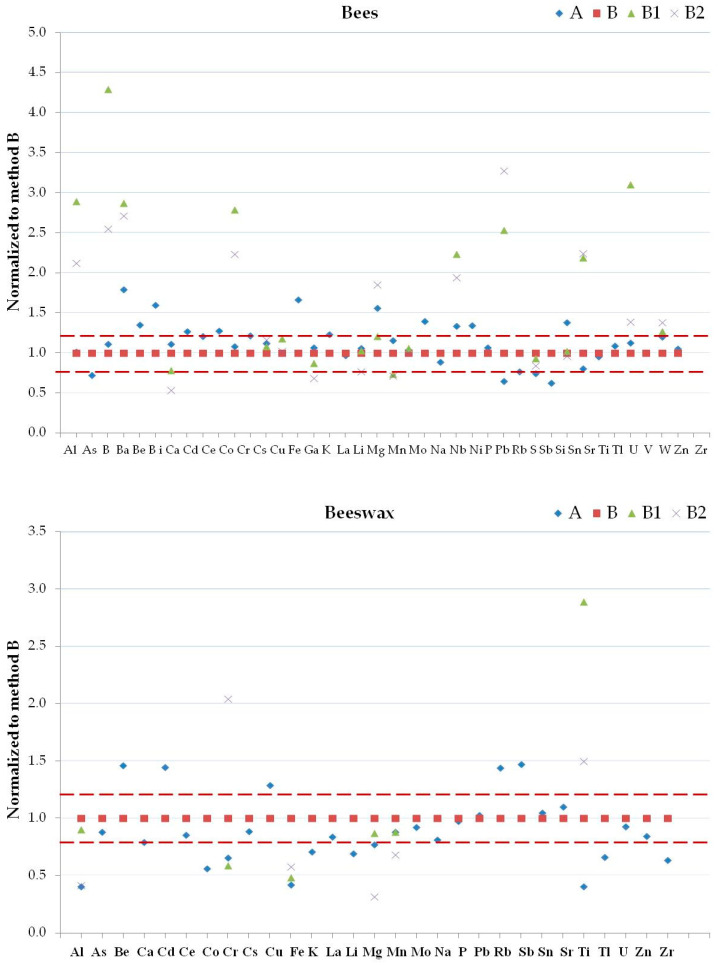
Normalized concentrations to results obtained by method B1 of detected elements in bees (top panel) and beeswax (bottom panel) samples by methods A, B, B1 and B2. Dashed lines are ±20%.

**Figure 3:**
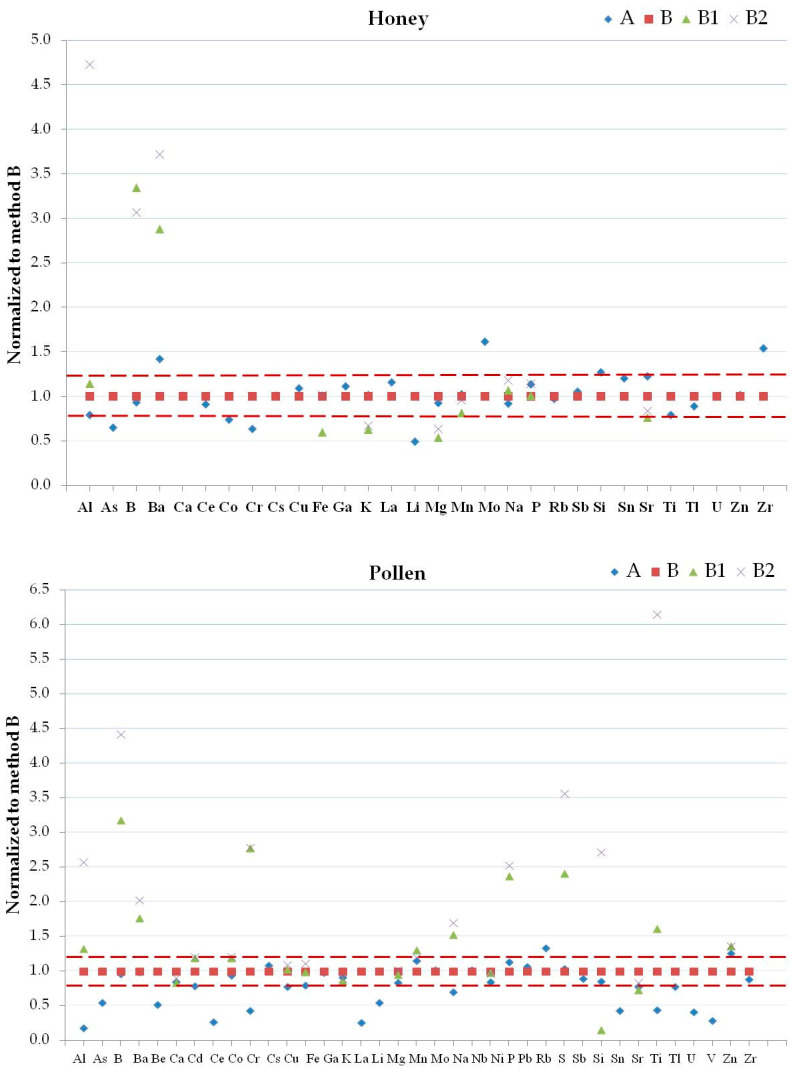
Normalized concentrations to results obtained by method B1 of detected elements in honey (top panel) and pollen (bottom panel) samples by methods A, B, B1 and B2. Dashed lines are ±20%.

**Figure 4:**
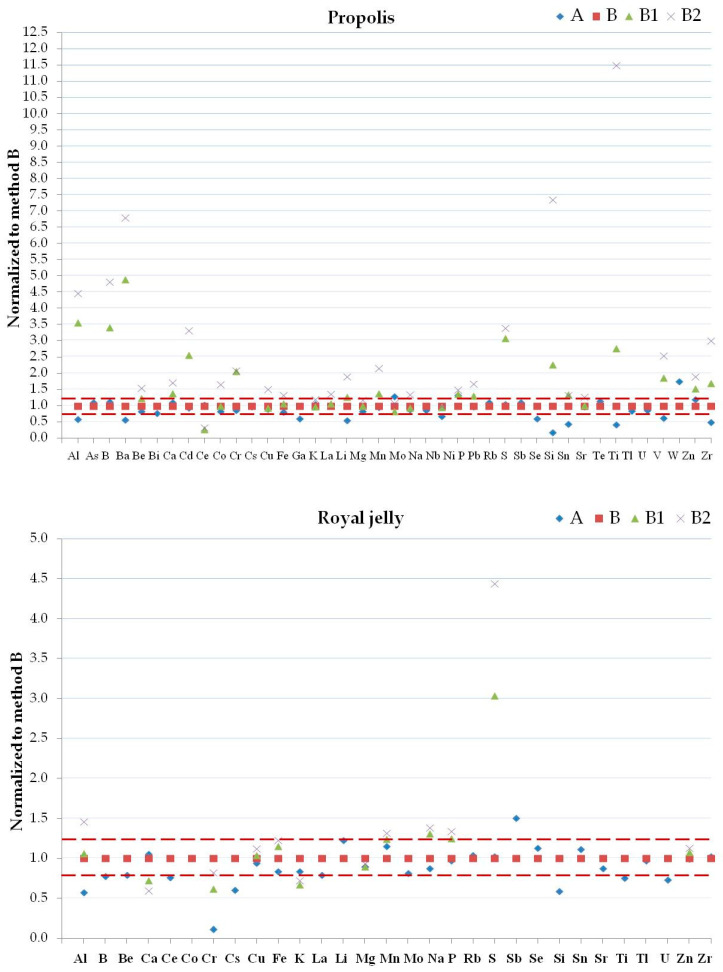
Normalized concentrations to results obtained by method B1 of detected elements in propolis (top panel) and royal jelly (bottom panel) samples by methods A, B, B1 and B2. Dashed lines are ±20%.

The method’s accuracy is very important for biomonitoring studies to control the quality of beehive products and to compare the results obtained from different samples. The variation of the elemental concentrations for each matrix must depend only on the variations of the environmental contamination and not on the selected method. The accuracy of the results allows for the assessment of the contamination in each matrix. For this purpose, a single homogeneous sample for each considered matrix was analyzed with the different digestion methods in order to estimate recovery and precision. Comparing the native data in the matrices (Figure 2, Figure 3 and Figure 4) normalized with respect to method B, the greatest differences observed between the results of Al, B, Ba, K, Mn, P, S, Ti, V, and Zn in bees; Al, Cr, Fe, Mg, and Ti in beeswax, Al, B, Ba, K, and Mg in honey, Al, B, Ba, Cr, Na, P, Si, Ti, and Zn in pollen, Al, B, Ba, Be, Ca, Cd, Ce, Co, Cr, Cu, Li, Mn, P, Pb, S, Si, Ti, V, Zn, and Zr in propolis, and Al, Na, and S in royal jelly were due to the use of different digestion mixtures and not to the use of different digestion systems. Se, and Te in bees, B, Ba, Bi, Ga, Nb, Ni, Se, Si, Te, V, and W in beeswax, Be, Bi, Cd, Nb, Ni, Pb, Se, Te, V, and W in honey, Bi, Se, Te, and W in pollen, As, Ba, Bi, Cd, Ga, Nb, Ni, Pb, Te, V, and W in royal jelly were not considered since they were always <LOD. Considering all the methods with the use of microwave oven, the reagent mixtures used in methods B1 and B2 allow a greater extraction of the elements in the different matrices compared to the method B. The extraction capacity of the digestion reagent mixtures depends on the chemical compounds present in the various analyzed matrices. The higher temperatures and pressure that occur with the use of the microwave oven compared to the water bath affected the content of Ba, Be, Bi, Ga, Mn, Nb, P, Pb, S, Sn, and Sr in bees, Al, Be, Cd, Co, Cr, Cu, Fe, Li, Rb, Sb, Ti, Tl, and Zr in beeswax, As, Ba, Cr, Li, Mo, and Zr in honey, Al, As, Be, Ce, Cr, La, Li, Rb, Ti, U, V, and Zn in pollen, Al, Ba, Ga, Li, Se, Si, Sn, Ti, V, W, and Zr in propolis, and Al, Cr, Cs, Sb, Si, and U in royal jelly. Tables S1–S6 show that methods A and B gave similar results for all the elements excluding Ga in bees, La, Mo, and Ti in honey, Be, Ce, La, Li, U, and V in pollen, Se in propolis, and Cr and Si in royal jelly. The total content of the following elements in the selected matrices can only be obtained by using aqua regia mixtures: Al, B, Ba, Cr, P, and S in bees; Ba, P, and Ti in beeswax; Al, and Ba in honey; Al, B, Ba, Cr, Cu, Fe, Na, S, Si, and Ti in pollen; Al, B, Ba, Be, Ca, Cd, Co, Cr, Li, Mg, Si, Sn, Ti, V, Zn, and Zr in propolis; Na, and P in royal jelly. The improvements and optimal recoveries of Al, Ba, Fe, and Sb from a variety of matrices upon the addition of HCl have been demonstrated [56,72]. However, treatment with HNO_3_ favors Cl elimination as nitrosyl chloride and minimizes the isobaric interferences in the case of some elements (As, Cr, Fe, Mn, Ni, Se and V) analyzed by ICP-MS [60,73].

In agreement with other studies [35], the procedure A is a good alternative to the procedure B because it limits sample manipulation, does not require the cleaning of sample vessels between analyses and allows 120 samples to be processed in 30 min. However, the content of some elements in specific matrices can be underestimated using the reactive digestion mixture HNO_3_/H_2_O_2_. The data reported in the present study aim to be a valuable help for choosing the most appropriated methods and analytical techniques for the determination of each element in bees and beehive products.

### 2.4. Analysis of Commercial Beehive Products

Commercial beehive samples were analyzed in order to demonstrate the applicability of the optimized methods. Concentrations were above the LODs for most of the elements, including Pb, showing that both methods A (Table 3) and B (Tables S10 and S11) can be used to determine the elemental composition of beehive products. Both methods A and B provided similar concentration data for the elements above the LODs. However, the average levels of some elements (La, Mo, and Ti in honey; Be, Ce, La, Li, U, and V in pollen; Se in propolis; Cr and Si in royal jelly) obtained with method B were higher. Methods A and B allow the elemental characterization of bees and beehive products, except for some elements that were underestimated with respect to the data acquired with methods B1 and B2, as reported in Section 2.3.4.

## 3. Materials and Methods

### 3.1. Instrumentation

A Bruker 820-MS quadrupole ICP-MS spectrometer (Bremen, Germany) equipped with a collision-reaction interface (CRI) and an Analytik Jena AG MicroMistTM glass nebulizer (0.4 mL min^−1^; Jena, Germany) was used for all the measurements. A radiofrequency power of 1.4 kW, plasma gas flow rate of 18.0 L min^−1^, auxiliary gas flow rate of 1.8 L min^−1^ and nebulizer gas flow rate of 1.0 L min^−1^ were used. The monitored isotopes (m/z) are shown in Table 1. The CRI with He and H_2_ (99.9995% purity; SOL Spa, Monza, Italy) as cell gases was used to quantify and remove polyatomic- and argon-based interference for As, Cr, Fe, Mn, Se, and V.

A Varian Vista MPX CCD Simultaneous ICP-OES spectrometer (Victoria, Mulgrave, Australia) in an axial configuration equipped with inert components (demountable torch with alumina injector, 1.8 mm, and PTFE injector holder; Sturman-Masters inert spray chamber, double pass, white Ertalyte; Agilent, Santa Clara, CA, United States) was used to determine the residual C content (RCC) of the final digests and all the selected elements. A radiofrequency power of 1.0 kW, plasma gas flow rate of 15.0 L min^−1^, auxiliary gas flow rate of 1.5 L min^−1^, and nebulizer gas flow rate of 0.75 L min^−1^ were used as operational conditions. Elements were detected at the wavelength that maximized the signal intensity and minimized spectral overlaps (Table 1).

Ar gas (99.9995% purity; SOL Spa, Monza, Italy) was used for plasma generation.

Analytical reagent-grade water with a resistivity of 18.2 MΩ cm was obtained with an Arioso Power I RO-UP Scholar UV water purification system from Human Corporation (Songpa-Ku, Seoul, Korea).

An Argo Lab WB12 water bath (Modena, Italy) with an electronic temperature control was used for open-vessel digestion (method A), as described in Section 3.3.1. A maximum temperature and pressure up to 95 °C (± 0.2 °C) and ~1 bar, respectively, were used for this system.

A Milestone Ethos1 Touch Control microwave system (Sorisole, Bergamo, Italy) equipped with six PTFE or 20 quartz vessels was used for closed-vessel microwave digestion (methods B, B1 and B2), as described in Section 3.3.2. The vessels were irradiated with a maximum power of 1000 W and all the experiments were carried out at the maximum temperature (180 °C) and pressures of ≤40 bar.

A Heto Power Dry LL1500 freeze dryer from Thermo Electron Corporation (Waltham, MA, USA) was employed for drying bee samples.

### 3.2. Reagents

Yttrium at 0.2 mg L^−1^ was used as the internal standard for ICP-OES and it was prepared from a standard stock solution (1000 ± 2 mg L^−1^; Panreac Química, Barcelona, Spain) [58]. Yttrium, Sc, Rh, In, and Th (1000 ± 5 mg L^−1^; Merck KGaA, Darmstadt, Germany) at 0.01 mg L^−1^ in a 1% (v/v) HNO_3_ multistandard solution were employed as internal standards for ICP-MS [58,74].

HNO_3_ (67–70%; super pure) from Carlo Erba Reagents S.r.l. (Milan, Italy), HCl (assay >36%; residue <3 mg L^−1^), HF (assay >40%; residue <2 mg L^−1^) and H_2_O_2_ (assay >30%) from Promochem, LGC Standards GmbH (Wesel, Germany) were used to prepare the standard and sample solution. All the reagents used were of analytical grade.

An apple leaf NIST 1515 was employed to evaluate the accuracy of the methods. The certified standard material was purchased from the National Institute of Standards and Technology (Gaithersburg, MD, USA).

ICP multielemental standard solutions of As, Al, Ba, Be, Bi, Cd, Cr, Cs, Cu, Ga, La, Li, Mn, Mo, Nb, Ni, Pb, Rb, Sb, Se, Sn, Te, Ti, Tl, U, V, W, and Zr at 1.000 ± 0.005 mg L^−1^, Ce and Co at 5.00 ± 0.03 mg L^−1^, Fe and Zn at 10.00 ± 0.05 mg L^−1^, P and Si at 50.00 ± 0.25 mg L^−1^, B and Sr at 55.00 ± 0.25 mg L^−1^, K, Mg, and Na at 500.0 ± 2.5 mg L^−1^, and Ca at 1000 ± 5 mg L^−1^ in 3% (v/v) HNO_3_, from Ultra Scientific/Agilent Technologies (North Kingstown, RI, USA), were used for the calibration procedure and spiked samples.

A multielemental standard solution of Ba, Be, Ce, Co, In, Pb, Mg, Tl, and Th (10.00 ± 0.05 mg L^−1^ in 2% HNO_3_) from Spectro Pure, Ricca Chemical Company (Arlington, TX, USA) was employed in order to select the best operating parameters for the ICP-MS analysis.

For the RCC assessment in the digested samples, a reference solution of 10,000 mg L^−1^ in C was prepared from anhydrous citric acid (assay >99.5%, ACS reagent; Sigma-Aldrich Chemie GmbH, Steinheim, Germany) in boiled deionized water, according to Muller et al., 2015 [49]. A standardized NaOH solution (0.5 mol L^−1^; assay >98% sodium hydroxide anhydrous pellets, RPE for analysis, ACS and ISO; Carlo Erba Reagents, Milan, Italy) was prepared for the determination of residual acidity in final digested samples by acid–base titration.

### 3.3. Sample Preparation Methods

Ten samples of commercially available beehive products were collected from local supermarkets in Rome (Central Italy). The set of samples comprised several brands and consisted of five multifloral honeys, two honeydews, one pollen, one royal jelly and one beeswax cream.

The bee samples used for the recovery experiments were collected directly from the hive. At least 10 bees per sample with a wet weight of ~2 g were brought to a stable dry weight after freeze-drying for 48 h and were finely grounded in a glass mortar. Specific portions of honeybees (30, 50, 100 and 200 mg) were analyzed to assess whether the weight of honeybees samples might be reduced. A mass of 200 mg was selected for the subsequent analyses.

A volume of 1 or 2 mL of concentrated HNO_3_ for each digestion method was selected considering the maximum volume of the digestion vessels and the minimum dilution of the samples, in order to have a final acidity of <5%, as recommended by the ICP-MS manual. The bee samples with mass of 200 mg and a reagent mixture ratio 1:2 of H_2_O_2_ and HNO_3_ were kept at maximum constant temperature of 95 °C for the water bath and 180 °C for the microwave oven, in accordance with previous studies [15,35,51]. Two commonly used digestion procedures with strong reagent mixtures [54,55,56], microwave aqua regia + H_2_O_2_ and microwave aqua regia + HF, were used for the total digestion of elements in bees and beehive products samples. The sample analyses were carried out in duplicate. Certified reference material (NIST SRM 1515; three replicates) and blank digests (ten replicates) were subjected to the same sample preparation. All tested analytical procedures are described below.

#### 3.3.1. Open-Vessel Water Bath-Assisted Digestion

A mass of ~200 mg for all samples was measured directly in an autosampler tube, 1 mL of 67% HNO_3_ and 0.5 mL of 30% H_2_O_2_ were then added in the same tube. Subsequently, tubes were heated to 95 ± 5 °C in a water bath for 30 min (method A). After digestion, the mixture was left to cool and the contents of the tubes were diluted to 10 or 20 mL with deionized water for ICP-OES or ICP-MS analyses, respectively.

#### 3.3.2. Closed-Vessel Microwave-Assisted Digestion

Weighed amounts (~200 mg) of all samples were transferred into the microwave vessels. Then, 1 mL 67% HNO_3_, 0.5 mL 30% H_2_O_2_, and 1.5 mL of deionized water (method B) were added to the quartz vessels, and 1 mL 30% H_2_O_2_ and 4 mL aqua regia (method B1) or 1 mL 40% HF and 4 mL aqua regia (method B2) were added to the PTFE vessels. Subsequently, vessels were heated to 180 °C with microwave energy (at a power of 1000 W) for 40 min. Cooled digests were transferred to the autosampler tubes. All digests by methods B and B1 were diluted to 10 or 20 mL with deionized water for the ICP-OES or ICP-MS analyses, respectively. The digests obtained with method B2 were diluted to a volume of 10 mL with deionized water for ICP-OES analysis.

### 3.4. Quality Assurance and Control

For the method validation, the selectivity, linearity, accuracy, LOD and LOQ were tested. According to the Eurachem Guide [75], accuracy, the closeness of agreement between a test result and the accepted reference value, is a parameter described by two contributions: the trueness bias or spike recovery and the precision. The accuracy was studied using a CRM and spiked samples for all the digestion methods or only for methods A and B, respectively.

The comparison between the obtained results and the certified values of the CRM was carried out using Z-scores [71]. These were calculated according to the following formula:

(1) $$({\bar{X}}_{\mathrm{found}}-{\bar{X}}_{\mathrm{certified}}/(\mathrm{SD}/\sqrt{n})$$

where ${\bar{X}}_{\mathrm{found}}$ is the result found by the analyst, ${\bar{X}}_{\mathrm{certified}}$ is the certified value, SD is the standard deviation, and n is the number of independent replicates. Z-scores smaller than 2 are usually considered acceptable, Z-scores between 2 and 3 are questionable, and Z-scores larger than 3 are not satisfactory.

For recovery experiments, a spike solution was added to the samples at two levels before the sample digestion. Each digestion batch contained a reagent blank (matrix samples) to allow background correction.

The LODs were calculated with three times the relative standard deviation percentage (RSD%) of ten method blanks multiplied by the background equivalent concentration (BEC)/100 [58], and the dilution factor used for sample preparation (Table S7). The LOQs were the lowest standard curve points that could be used for quantification (LLOQs). Together, the LLOQ and upper LOQ (ULOQ) define the linearity range.

At regular intervals (every 20 samples) during all analyses, an intermediate calibration standard was analyzed as a sample to monitor the instrument drift. A maximum percentage drift of ± 10% was considered acceptable for all the elements. Furthermore, calibration blanks (3% HNO_3_) were frequently analyzed alongside samples to check any loss or cross contamination. Blanks were prepared by performing the full analytical procedure without samples.

The matrix effects on the sample uptake and nebulization were monitored by an internal standardization with Y for ICP-OES, and Y, Sc, Rh, In and Th for ICP-MS, and measurements were automatically corrected by the respective ICP software. Yttrium was not used as an internal standard for the ICP-MS analyses of the propolis samples because it is contained in the propolis in a concentration of ~0.25 mg kg^−1^ (~0.0025 mg L^−1^ in digests). This concentration was negligible for ICP-OES analyses because Y was added to the samples as an internal standard in a concentration of 0.2 mg L^−1^; therefore, in this case, Y was used as the internal standard for the propolis samples.

### 3.5. Statistical Analysis

All statistical calculations were made by the SPSS software package (IBM SPSS Statistics 25 software; IBM Corp., Armonk, NY, USA). Values < LOD were designated as LOD/2 [76]. The studies of significant differences were carried out by Mann–Whitney U or Kruskal–Wallis tests with pairwise post-hoc tests [77]. The difference in the results was considered statistically significant for *p*-values < 0.05.

## 4. Conclusions

Unfortunately, the validation of methods and procedures used for the analysis of bees and beehive products is difficult because no CRMs of these matrices are available. Instead, the trueness of the methods and procedures applied is commonly checked by the analysis of different CRMs or by the recovery tests with samples spiked with known amounts of elements. It is worth noting that the study of elemental recovery in bees and beehive products may not allow for the evaluation of the efficiency of digestion procedures in decomposing the sample matrix. This in fact may not have the ability to readily absorb aqueous solutions containing known amounts of elements that should be released with only a complete digestion of the sample. In the present study, it is highlighted how each element in a specific matrix responds differently to the different sample treatment procedures used, thus allowing one to choose a specific method accordingly for the needs and purpose of the analysis. In screening analyses and biomonitoring studies, method A was a faster and a good alternative compared to microwave-assisted acid digestion for the determination of all the analyzed elements. Both the methods with mixture HNO_3_/H_2_O_2_ (methods A and B) showed an acceptable accuracy for all the analyzed elements, and low levels of detection for trace elements including Pb. Considering all the tested methods, to have total levels of some elements (such as Al, B, Ba, Cr, P, and S in bees; Ba, P, and Ti in beeswax; Al, and Ba in honey; Al, B, Ba, Cr, Cu, Fe, Na, S, Si, and Ti in pollen; Al, B, Ba, Be, Ca, Cd, Co, Cr, Li, Mg, Si, Sn, Ti, V, Zn, and Zr in propolis; Na, and P in royal jelly) is necessary to use an aqua regia mixture. This is very important for evaluating the quality of products and for preserving human health and the environment.

## Figures and Tables

**Table 1:** Limit of determination ^a^ (mg kg^−1^) and linearity range for each element in bees and beehive products by inductively coupled plasma spectrometry.

Isotope/Element ^b^	Internal Standard ^c^				LLOQ–ULOQ ^d^ mg kg^−1^			
		LOD_A_	LOD_B_	LOD_B1_	N ^e^	Honey and Beeswax	N ^e^	Bees, Pollen, Propolis, and Royal Jelly
27Al	45Sc	0.06	0.02	0.02	4	0.5–5	5	1.05–21
75As ^f^	79Y	0.001	0.001	-	6	0.05–2	6	0.05–2
11B	45Sc	0.2	0.1	0.1	5	2.75–55	5	2.75–55
137Ba	115In	0.4	0.2	0.2	5	0.2–5	4	7–100
9Be	45Sc	0.00002	0.00002	0.00002	7	0.05–5	7	0.05–5
209Bi	232Th	0.00004	0.00006	0.00005	6	0.05–2	6	0.05–2
44Ca	79Y	11	5	14	4	100–1000	4	1000–10000
112Cd	115In	0.00004	0.00001	0.00001	7	0.05–110	7	0.05–110
140Ce	115In	0.00006	0.00004	0.0001	5	0.25–5	5	0.25–5
59Co	45Sc	0.0005	0.0004	0.0008	6	0.25–10	6	0.25–10
52Crf	79Y	0.001	0.0008	-	7	0.05–5	4	1–10
133Cs	115In	0.00004	0.00002	0.00001	7	0.05–5	7	0.05–5
65Cu	79Y	0.003	0.002	0.002	6	0.05–2	9	0.1–100
56Fe	79Y	0.02	0.01	0.01	4	1–10	9	2.5–5000
71Ga	79Y	0.00005	0.00001	0.00001	4	0.1–1	4	0.1–1
39K	45Sc	5	3	0.7	6	50–2500	6	50–2500
139La	115In	0.00004	0.00006	0.00005	7	0.05–5	7	0.05–5
7Li	45Sc	0.0008	0.0008	0.0005	7	0.05–5	7	0.05–5
24Mg	45Sc	0.9	0.3	1.0	5	100–2500	5	50–1000
55Mn	79Y	0.002	0.002	0.003	4	0.2–2	6	5.2–200
98Mo	103Rh	0.0003	0.0001	0.0001	7	0.05–110	7	0.05–110
23Na	45Sc	0.6	0.4	0.3	5	100–2500	7	25–2000
93Nb	103Rh	0.00001	0.000007	0.00002	5	0.05–1	5	0.05–1
60Ni	45Sc	0.002	0.003	0.003	6	0.05–2	7	0.2–20
31P	45Sc	0.7	1	0.7	5	5–100	4	1100–5000
208Pb	232Th	0.001	0.001	0.001	7	0.05–5	4	7–100
85Rb	79Y	0.0003	0.0003	0.00007	5	0.05–1	4	20–200
121Sb	115In	0.0004	0.0002	0.0001	7	0.05–5	7	0.05–5
76Se	79Y	0.02	0.007	0.01	4	0.2–2	4	0.2–2
28Si	45Sc	9	8	10	4	10–100	4	50–500
118Sn	115In	0.0002	0.0001	0.0001	7	0.05–5	7	0.05–5
88Sr	79Y	0.008	0.02	0.02	5	5.5–110	5	5.5–110
125Te	115In	0.0003	0.0003	0.0002	5	0.05–1	5	0.05–1
49Ti	45Sc	0.002	0.003	0.0007	9	0.05–50	9	0.05–50
205Tl	232Th	0.00006	0.00003	0.00001	5	0.05–1	5	0.05–1
238U	232Th	0.00001	0.00001	0.00001	7	0.05–5	7	0.05–5
51V	79Y	0.0003	0.0006	0.00003	6	0.1–5	4	1–10
182W	232Th	0.0003	0.0002	0.00006	7	0.05–5	7	0.05–5
66Zn	79Y	0.09	0.04	0.09	5	2–50	4	70–1000
90Zr	79Y	0.0001	0.00009	0.0001	6	0.05–2	6	0.05–2

^a^ LOD_A_, LOD_B_, and LOD_B1_ are the limits of determination for methods A, B, and B1, respectively. ^b^ Isotopes are reported for all the analyzed elements by inductively coupled plasma mass spectrometry (ICP-MS). ^c^ Rh replaces Y for propolis samples. ^d^ LLOQ, lower limit of quantification; ULOQ upper limit of quantification. ^e^ N, number of calibration points for honey and beeswax or bees, pollen, propolis and royal jelly. ^f^ As and Cr in digests obtained by method B1 are determined with inductively coupled plasma optical emission spectrometry (ICP-OES).

**Table 2:** Summary of precision (repeatability as percent coefficient of variation intrarun (%CVr) and reproducibility as %CVR inter-run), and percent recovery (%R) ranges for each element in working bee and beehive products applying two different digestion methods prior to analysis by ICP-MS and ICP-OES (for S).

Isotope/Element ^a^	Method A			Method B		
	%CVr Intraday (*n* = 3)	%CVR Interday (*n* = 9)	%R (*n* = 3)	%CVr Intraday (*n* = 3)	%CVR Interday (*n* = 9)	%R (*n* = 3)
^27^Al	1.8–24	1.5–25	96–126	5.4–27	15–25	97–124
^75^As	1.8–21	17–27	88–124	5.0–29	17–30	90–119
^11^B	0.2–24	6.5–19	84–102	2.2–32	7.6–25	86–100
^137^Ba	0.6–23	11–25	103–122	1.2–21	17–30	101–112
^9^Be	0.9–13	8.2–24	86–97	14–22	21–30	90–101
^209^Bi	1.8–17	11–21	85–107	3.1–25	19–31	86–110
^44^Ca	1.4–12	7.2–25	79–127	5.7–26	14–26	82–127
^112^Cd	0.7–19	9.1–25	83–100	1.5–22	19–30	83–104
^140^Ce	1.1–19	8.7–26	96–123	1.0–25	10–30	98–119
^59^Co	0.8–13	1.1–25	81–105	0.4–27	9.2–27	84–102
^52^Cr	0.7–19	2.0–22	91–101	8.9–26	20–31	94–107
^133^Cs	0.7–6.4	9.1–25	99–123	3.3–14	3.3–30	99–119
^65^Cu	1.3–23	4.6–23	82–121	1.1–18	5.4–28	87–111
^56^Fe	0.5–7.7	3.6–13	88–120	0.9–25	5.9–25	92–116
^71^Ga	1.2–23	13–24	83–130	1.1–25	7.4–31	86–130
^39^K	0.9–22	1.3–21	87–124	0.8–10	6.7–13	87–121
^139^La	1.2–10	5.0–24	90–114	0.5–24	8.1–29	92–114
^7^Li	0.2–11	3.8–24	90–118	1.5–23	14–21	90–120
^24^Mg	0.01–9.0	5.2–25	82–108	0.5–19	7.0–23	82–110
^55^Mn	0.4–17	12–25	84–125	0.7–24	3.7–24	86–115
^98^Mo	0.3–6	10–25	95–117	1.9–25	1.9–25	94–114
^23^Na	0.6–6.7	4.9–21	80–125	2.2–17	6.5–26	84–122
^93^Nb	1.5–20	8.0–24	85–122	3.6–22	11–27	86–116
^60^Ni	0.9–17	13–22	83–97	0.4–23	8.6–29	82–99
^31^P	0.5–11	4.2–20	80–122	0.5–23	5.7–25	81–123
^208^Pb	1.6–23	12–24	80–121	2.7–24	16–27	86–116
^85^Rb	3.6–11	4.5–25	96–120	0.8–23	7.3–30	92–117
S	7.5–9.1	6.8–11	96–110	1.7–10	4.1–11	97–111
^121^Sb	0.2–17	11–26	81–100	13–23	15–27	84–98
^76^Se	0.4–18	16–26	83–108	3.4–24	13–30	82–102
^28^Si	1.4–15	4.2–26	101–126	1.8–21	4.1–28	98–122
^118^Sn	2.0–14	5.7–26	81–101	2.7–23	15–31	86–101
^88^Sr	1.3–15	0.6–25	89–121	0.5–24	15–34	84–119
^125^Te	0.7–15	7.0–26	81–89	3.0–17	27–30	81–90
^49^Ti	1.2–22	6.8–18	95–128	0.2–19	3.5–30	96–118
^205^Tl	1.6–19	13–20	94–124	1.0–19	11–24	96–120
^238^U	1.7–21	9.8–29	87–110	1.9–21	15–26	87–110
^51^V	0.3–19	9.4–25	83–111	1.2–22	10–30	86–106
^182^W	1.8–7	19–26	84–113	0.6–15	11–30	84–116
^66^Zn	0.3–24	5.2–25	88–126	1.3–21	10–29	86–124
^90^Zr	1.2–21	11–25	90–112	2.3–17	13–31	92–114

^a^ Isotopes were reported for all the analyzed elements by ICP-MS. Sulfur was determined by ICP-OES.

**Table 3:** Concentrations (mg Kg^−1^) of each element in some commercial apiary products obtained by method A.

Element	Honey 1		Honey 2		Honey 3		Honey 4		Honey 5		Honeydew 1		Honeydew 2		Beeswax		Pollen		Royal Jelly	
	M	SD	M	SD	M	SD	M	SD	M	SD	M	SD	M	SD	M	SD	M	SD	M	SD
Al	0.09	0.02	0.187	0.078	6.33	0.43	2.48	0.22	0.153	0.027	0.439	0.035	1.65	0.03	<LOD	-	3.68	0.29	0.054	0.025
As	<LOD	-	<LOD	-	<LOD		<LOD		<LOD	-	<LOD	-	<LOD	-	<LOD	-	0.012	0.011	<LOD	-
B	6.56	0.32	0.697	0.045	3.60	0.25	3.99	0.03	7.75	0.16	4.54	0.10	7.33	0.46	<LOD	-	5.73	0.67	1.37	0.16
Ba	0.083	0.007	<LOD	-	1.93	0.08	0.627	0.043	0.0522	0.0053	0.113	0.011	0.891	0.061	<LOD	-	2.15	0.42	<LOD	-
Be	<LOD	-	<LOD	-	0.00091	0.00016	0.00117	0.00019	<LOD	-	<LOD	-	0.00183	0.00032	<LOD	-	0.00052	0.00037	<LOD	-
Bi	<LOD	-	<LOD	-	0.000516	0.000042	<LOD	-	<LOD	-	0.00057	0.00013	0.000270	0.000069	<LOD	-	<LOD	-	<LOD	-
Ca	91	6	38.6	8.7	181	24	154	21	51.1	6.8	50	12	118	8	<LOD	-	547	103	165	43
Cd	0.000218	0.000058	<LOD	-	0.00104	0.00034	<LOD	-	<LOD	-	0.00115	0.00022	0.00087	0.00010	<LOD	-	0.0642	0.0063	<LOD	-
Ce	0.00117	0.00015	0.000372	0.000087	0.0145	0.0012	0.0187	0.0012	0.00096	0.00025	0.00234	0.00017	0.0419	0.0018	0.00098	0.00023	0.0055	0.0026	0.000850	0.0002
Co	0.00630	0.00007	0.00130	0.00031	0.0107	0.0005	0.0107	0.0005	0.00174	0.00030	0.0176	0.0008	0.0241	0.0020	<LOD	-	0.158	0.017	0.00123	0.00023
Cr	0.00561	0.00074	0.0063	0.0017	0.0123	0.0016	0.0127	0.0033	<LOD	-	0.0146	0.0022	0.0587	0.0055	0.0204	0.0060	0.0547	0.0061	0.0340	0.0026
Cs	0.00443	0.00034	0.00062	0.00010	0.787	0.045	0.565	0.029	0.00245	0.00065	0.00365	0.00013	0.547	0.037	0.000292	0.000091	0.0142	0.0034	0.00063	0.00013
Cu	0.261	0.013	0.0623	0.0073	0.747	0.039	1.01	0.02	0.153	0.010	1.97	0.02	3.30	0.14	<LOD	-	5.82	0.89	4.26	0.41
Fe	2.93	0.41	1.81	0.19	2.68	0.31	4.28	0.96	0.58	0.13	2.54	0.25	4.50	0.35	0.31	0.15	24.0	8.3	9.23	0.75
Ga	0.00247	0.00035	0.00240	0.00029	0.042	0.011	0.0140	0.0012	0.00173	0.00035	0.00224	0.00048	0.0201	0.0025	<LOD	-	0.0436	0.0072	<LOD	-
K	1340	24	89	2	4661	341	3708	36	732	8	6520	46	2399	157	<LOD	-	3703	592	2520	260

## Supplementary Materials

The following are available online, Figure S1, Tables S1–S11.
