# Supplementary material for: Effectiveness of Different Sample Treatments for the Elemental Characterization of Bees and Beehive Products
Source: Molecules. 2020 Sep 17;25(18):4263. doi: 10.3390/molecules25184263 (PMC7570605; doi:10.3390/molecules25184263)
Supplement: Supplementary file 1 [file molecules-25-04263-s001.pdf]

Supplementary Materials

Effectiveness of different sample treatments for the elemental characterization of bees and beehive products

Maria Luisa Astolfi [1,*], Marcelo Enrique Conti [2], Elisabetta Marconi [3], Lorenzo Massimi [1] and Silvia Canepari [1]

[1] Department of Chemistry, Sapienza University, Piazzale Aldo Moro 5, I-00185 Rome, Italy; marialuisa.astolfi@uniroma1.it
[2] Department of Management, Sapienza University, Via del Castro Laurenziano 9, I-00161 Rome, Italy
[3] Department of Public Health and Infectious Diseases, Sapienza University, Piazzale Aldo Moro 5, I-00185 Rome, Italy
* Correspondence: marialuisa.astolfi@uniroma1.it

Table S1. Element concentrations (mg kg^{-1}) in working bee samples applying four digestion methods prior to analysis by ICP-MS and ICP-OES.

Element	N[a]	Measured concentration[b]							
		Method A		Method B		Method B1		Method B2	
		M	SD	M	SD	M	SD	M	SD
**Al**	18	**17.7[c]**	**4.0**	**17.4[d]**	**3.5**	**50.4[c,d]**	**5.2**	37	12
**As**	13	0.0182	0.0096	0.025	0.015	<2	-	<2	-
**B**	18	**13.7[c]**	**3.9**	**12.3[d]**	**4.0**	**52.8[c,d]**	**2.1**	31.3	5.2
**Ba**	18	2.45	0.48	**1.36[c,d]**	**0.63**	**3.9[c]**	**1.1**	**3.71[d]**	**0.38**
**Be**	13	0.00397	0.00066	0.0029	0.0013	<0.02	-	<0.02	-
**Bi**	13	0.0048	0.0025	0.00301	0.00084	<0.4	-	<0.1	-
**Ca**	18	**818[c]**	**190**	737	130	578	33	**395[c]**	**40**
**Cd**	12	0.0070	0.0027	0.0055	0.0011	<0.06	-	<0.02	-
**Ce**	12	0.094	0.023	0.078	0.032	<0.1	-	<0.4	-
**Co**	14	0.046	0.015	0.036	0.010	<0.09	-	<0.1	-
**Cr**	18	**0.047[c]**	**0.009**	**0.043[d]**	**0.010**	**0.121[c,d]**	**0.008**	<0.1	-
**Cs**	12	0.0271	0.0060	0.022	0.010	-	-	-	-
**Cu**	18	7.83	0.94	6.98	0.74	7.5	1.2	8.13	0.69
**Fe**	18	49.5	5.9	49.4	13.0	58.4	9.7	50.4	7.2
**Ga**	12	**0.070[c]**	**0.013**	**0.042[c]**	**0.024**	<1	-	<0.4	-
**K**	18	**5109[c]**	**435**	4801	719	4186	347	**3287[c]**	**302**
**La**	13	0.048	0.012	0.039	0.015	<0.07	-	<0.05	-
**Li**	12	0.0179	0.0035	0.0185	0.0084	<0.1	-	<0.1	-
**Mg**	18	**411[c]**	**50**	388	87	400	27	**298[c]**	**6**
**Mn**	18	13.5	6.1	8.6	1.9	10.5	4.3	16.0	3.1
**Mo**	18	**0.176[c]**	**0.015**	0.152	0.039	0.112	0.011	**0.109[c]**	**0.011**
**Na**	18	274	26	264	24	280	15	267	14
**Nb**	12	0.0087	0.0025	0.0063	0.0026	<0.1	-	<0.3	-
**Ni**	12	0.057	0.020	0.064	0.021	<0.4	-	<0.6	-
**P**	18	1980	726	**1484[c]**	**186**	**3322[c]**	**294**	2890	76
**Pb**	15	0.141	0.043	0.105	0.035	<0.2	-	<0.2	-
**Rb**	15	13.0	1.4	12.2	3.5	0.69	0.25	<2	-
**S[e]**	10	**462[c]**	**40**	713	105	1805	168	**2334[c]**	**164**
**Sb**	12	0.0215	0.0035	0.028	0.030	<0.7	-	<0.5	-
**Se**	7	<0.02	0.0065	0.0082	0.0046	<4	-	<4	-
**Si**	18	62	11	83	27	78	16	70	45
**Sn**	13	0.085	0.035	0.135	0.052	<0.1	-	<0.1	-
**Sr**	18	3.3	1.4	2.4	1.1	2.47	0.39	2.33	0.04
**Te**	7	0.00058	0.00056	0.00078	0.00077	<1	-	<0.6	-
**Ti**	18	0.88	0.22	1.09	0.74	2.39	0.39	2.45	0.58
**Tl**	12	0.068	0.032	0.072	0.045	<4	-	<3	-
**U**	12	0.00366	0.00069	0.0034	0.0014	<3	-	<3	-
**V**	16	0.053	0.014	0.047	0.015	0.146	0.028	<1	-
**W**	12	0.037	0.018	0.037	0.020	<1	-	<1	-
**Zn**	18	33.6	22.0	27.9	3.5	35	13	38.5	7.3
**Zr**	13	0.052	0.015	0.049	0.014	<0.3	-	<0.1	-

[a] N, number of valid data. [b] M, mean; SD, standard deviation. Digests obtained by methods A or B were analyzed with ICP-MS, and digests obtained by methods B1 or B2 were analysed with ICP-OES. [c,d] Results marked in bold were considered statistically significant for p-values <0.05. Same superscript letters within rows are significantly different. [e] S was determined with ICP-OES.

Table S2. Element concentrations (mg kg^{-1}) in working beeswax samples applying four digestion methods prior to analysis by ICP-MS and ICP-OES.

Element	N[a]	Measured concentration[b]							
		Method A		Method B		Method B1		Method B2	
		M	SD	M	SD	M	SD	M	SD
**Al**	16	0.75	0.34	1.8	1.5	1.65	0.56	0.76	0.46
**As**	11	0.0041	0.0036	0.0047	0.0035	<2	-	<2	-
**B**	3	0.22	0.31	0.28	0.34	<13	-	<8	-
**Ba**	10	<0.4		<0.2		1.12	0.39	1.22	0.97
**Be**	10	0.00053	0.00046	0.00036	0.00016	<0.02	-	<0.02	-
**Bi**	10	<0.4		<0.1		<0.4	-	<0.1	-
**Ca**	13	155	36	196	56	161	26	<80	-
**Cd**	11	0.0031	0.0019	0.00215	0.00047	<0.06	-	<0.02	-
**Ce**	11	0.0085	0.0014	0.0099	0.0017	<0.1	-	<0.4	-
**Co**	10	0.0025	0.0018	0.0043	0.0032	<0.09	-	<0.1	-
**Cr**	17	0.41	0.51	0.62	0.26	0.37	0.19	1.26	0.77
**Cs**	11	0.00073	0.00044	0.00082	0.00069	-	-	-	-
**Cu**	11	0.204	0.046	0.159	0.060	<0.4	-	<0.3	-
**Fe**	17	3.7	3.0	8.8	4.4	4.2	1.8	5.1	3.8
**Ga**	7	0.0145	0.0156	0.0043	0.0086	<1	-	<0.4	-
**K**	10	15.1	6.6	21	24	<140	-	<100	-
**La**	11	0.0042	0.0006	0.0050	0.0021	<0.07	-	<0.05	-
**Li**	11	0.0036	0.0016	0.0051	0.0039	<0.1	-	<0.1	-
**Mg**	16	3.8	1.5	4.9	2.1	4.24	0.35	<2	-
**Mn**	17	0.277	0.090	0.31	0.14	0.277	0.025	0.21	0.15
**Mo**	11	0.0034	0.0028	0.0037	0.0013	<0.09	-	<0.1	-
**Na**	11	10.2	5.7	12.5	4.4	<10	-	<12	-
**Nb**	9	0.00029	0.00036	0.00016	0.00020	<0.1	-	<0.3	-
**Ni**	11	0.028	0.028	0.087	0.048	<0.4	-	<0.6	-
**P**	13	10.6	6.8	10.9	7.4	<20	-	<20	-
**Pb**	13	0.214	0.039	0.208	0.027	<0.2	-	<0.2	-
**Rb**	13	0.028	0.021	0.0195	0.0087	0.73	0.38	<2	-
**S[e]**	5	<20		<20		90	45	35	21
**Sb**	11	0.0050	0.0025	0.00339	0.00048	<0.7	-	<0.5	-
**Se**	1	<0.02		<0.007		<4	-	<4	-
**Si**	2	<9		<8		<15	-	<15	-
**Sn**	14	0.021	0.014	0.0198	0.0060	0.28	0.20	0.11	0.11
**Sr**	14	0.40	0.14	0.36	0.18	0.171	0.011	<0.3	-
**Te**	5	0.0009	0.0011	<0.003		<1	-	<0.6	-
**Ti**	16	**0.030[c]**	**0.016**	0.075	0.047	**0.22[c]**	**0.13**	0.112	0.058
**Tl**	11	0.00052	0.00022	0.00078	0.00029	<4	-	<3	-
**U**	11	0.00134	0.00039	0.00144	0.00021	<3	-	<3	-
**V**	4	0.0013	0.0020	0.0015	0.0025	<0.1	-	<0.1	-
**W**	1	<0.0003		<0.0002		<1	-	<1	-
**Zn**	14	3.2	1.4	3.8	1.1	4.34	0.78	<3	-
**Zr**	11	0.0040	0.0032	0.0063	0.0040	<0.3	-	<0.1	-

[a] N, number of valid data. [b] M, mean; SD, standard deviation. Digests obtained by methods A or B were analyzed with ICP-MS, and digests obtained by methods B1 or B2 were analysed with ICP-OES. [c,d] Results marked in bold were considered statistically significant for p-values <0.05. Same superscript letters within rows are significantly different. [e] S was determined with ICP-OES.

Table S3. Element concentrations (mg kg^{-1}) in working honey samples applying four digestion methods prior to analysis by ICP-MS and ICP-OES.

Element	N[a]	Measured concentration[b]							
		Method A		Method B		Method B1		Method B2	
		M	SD	M	SD	M	SD	M	SD
Al	14	**0.48[c]**	**0.13**	0.61	0.14	0.701	0.070	**2.90[c]**	**0.27**
As	8	0.0037	0.0018	0.0057	0.0038	<2	-	<2	-
B	14	4.67	0.56	5.0	1.0	16.8	3.1	15.4	0.3
Ba	12	0.63	0.34	**0.45**	**0.39[c]**	1.28	0.22	**1.66[c]**	**0.11**
Be	0	<0.00002		<0.00002		<0.02	-	<0.02	-
Bi	3	<0.00004		<0.00006		<0.4	-	<0.1	-
Ca	8	85	17	84	17	<140	-	<80	-
Cd	3	0.00031	0.00050	0.00012	0.00010	<0.06	-	<0.02	-
Ce	8	0.00124	0.00015	0.00136	0.00024	<0.1	-	<0.4	-
Co	8	0.00142	0.00039	0.00192	0.00035	<0.09	-	<0.1	-
Cr	8	0.14	0.16	0.22	0.33	<0.1	-	<0.1	-
Cs	8	0.0188	0.0033	0.0187	0.0031	-	-	-	-
Cu	9	0.157	0.008	0.144	0.033	<0.4	-	<0.3	-
Fe	14	1.45	0.87	1.44	0.91	0.863	0.084	1.46	0.36
Ga	8	0.025	0.012	0.0220	0.0075	<1	-	<0.4	-
K	14	752	99	741	80	462	51	497	13
La	8	**0.000615[c]**	**0.000028**	**0.000532[c]**	**0.000036**	<0.07	-	<0.05	-
Li	8	0.0020	0.0015	0.0041	0.0016	<0.1	-	<0.1	-
Mg	14	21.8	2.2	**23.5[c]**	**4.1**	**12.6[c]**	**0.5**	14.9	0.8
Mn	14	0.248	0.030	0.243	0.019	0.20	0.012	0.231	0.002
Mo	8	**0.00416[c]**	**0.00086**	**0.00258[c]**	**0.00011**	<0.09	-	<0.1	-
Na	14	28.0	1.6	30.5	7.9	32.5	1.4	35.9	1.9
Nb	6	0.00051	0.00044	0.000029	0.000050	<0.1	-	<0.3	-
Ni	0	<0.002		<0.003		<0.4	-	<0.6	-
P	14	58	19	51	12	52	5.0	58.5	1.9
Pb	0	<0.001		<0.001	0.037	<0.2	-	<0.2	-
Rb	9	4.87	0.64	5.02	0.82	<0.6	-	<2	-
S[e]	5	<20		23.9	0.4	<52	-	48.6	1.3
Sb	7	0.0037	0.0030	0.0035	0.0032	<0.7	-	<0.5	-
Se	0	<0.02		<0.007		<4	-	<4	-
Si	8	57.0	6.6	44.8	6.8	<15	-	<15	-
Sn	10	0.00362	0.00056	0.00301	0.00091	<0.1	-	<0.1	-
Sr	14	0.93	0.33	0.76	0.28	0.580	0.056	0.633	0.033
Te	4	0.00052	0.00039	<0.0003		<1	-	<0.6	-
Ti	10	**0.0110[c]**	**0.0008**	**0.0138[c]**	**0.0008**	<0.05	-	<0.1	-
Tl	8	0.0101	0.0017	0.0113	0.0030	<4	-	<3	-
U	7	0.000048	0.000014	0.000048	0.000057	<3	-	<3	-
V	6	0.0037	0.0038	0.0012	0.0010	<0.1	-	<0.1	-
W	5	0.00086	0.00074	0.00038	0.00024	<1	-	<1	-
Zn	8	0.98	0.24	0.97	0.18	<3	-	<3	-
Zr	8	0.00273	0.00059	0.00178	0.00029	<0.3	-	<0.1	-

[a] N, number of valid data. [b] M, mean; SD, standard deviation. Digests obtained by methods A or B were analyzed with ICP-MS, and digests obtained by methods B1 or B2 were analysed with ICP-OES. [c,d] Results marked in bold were considered statistically significant for p-values <0.05. Same superscript letters within rows are significantly different. [e] S was determined with ICP-OES.

Table S4. Element concentrations (mg kg^{-1}) in working bee pollen samples applying four digestion methods prior to analysis by ICP-MS and ICP-OES.

Element	N[a]	Measured concentration[b]							
		Method A		Method B		Method B1		Method B2	
		M	SD	M	SD	M	SD	M	SD
Al	18	**3.3[c,d]**	**1.5**	18.2	2.1	**24.0[c]**	**3.4**	**47[d]**	**12**
As	12	0.0140	0.0070	0.0258	0.0087	<2	-	<2	-
B	18	**9.2[c]**	**3.0**	**9.6[d]**	**2.7**	**30.5[c]**	**3.1**	**42.4[d]**	**9.2**
Ba	18	**2.05[c]**	**0.57**	2.09	0.35	3.68	0.31	**4.23[c]**	**0.59**
Be	12	**0.00059[c]**	**0.00030**	**0.00114[c]**	**0.00029**	<0.02	-	<0.02	-
Bi	9	0.00045	0.00065	0.00055	0.00084	<0.4	-	<0.1	-
Ca	18	726	202	863	256	715	66	778	49
Cd	18	0.048	0.014	0.061	0.015	0.073	0.011	0.075	0.013
Ce	12	**0.0106[c]**	**0.0037**	**0.0395[c]**	**0.0057**	<0.1	-	<0.4	-
Co	18	0.151	0.020	0.161	0.015	0.191	0.031	0.193	0.026
Cr	18	**0.0173[c,d]**	**0.0063**	0.040	0.032	**0.112[c]**	**0.037**	**0.113[d]**	**0.024**
Cs	12	0.041	0.027	0.038	0.010	-	-	-	-
Cu	18	**5.7[c]**	**1.4**	7.3	1.8	7.44	0.24	**7.96[c]**	**0.21**
Fe	18	**29.6[c]**	**4.4**	37.3	2.2	36.8	4.6	**41.5[c]**	**7.1**
Ga	12	0.050	0.031	0.051	0.026	<1	-	<0.4	-
K	18	4755	973	5199	678	4470	281	4641	97
La	13	**0.0048[c]**	**0.0016**	**0.0186[c]**	**0.0029**	0.079	0.085	<0.05	-
Li	12	**0.0164[c]**	**0.0031**	**0.0302[c]**	**0.0083**	<0.1	-	<0.1	-
Mg	18	392	109	469	33	447	12	478	21
Mn	18	32	11	27.5	9.1	35.8	5.2	33.0	4.6
Mo	13	0.0200	0.0071	0.0197	0.0083	<0.09	-	<0.1	-
Na	18	**28.4[c]**	**8.7**	40.8	11.0	62.2	2.3	**69.3[c]**	**0.1**
Nb	12	0.00116	0.00062	0.00114	0.00042	<0.1	-	<0.3	-
Ni	18	1.43	0.44	1.69	0.36	1.66	0.11	1.64	0.19
P	18	1751	625	1554	41	3688	320	3917	75
Pb	12	0.028	0.035	0.026	0.012	<0.2	-	<0.2	-
Rb	10	7.2	3.8	5.4	1.0	<0.6	-	<2	-
S[e]	10	735	67	**714[c]**	**82**	1718	132	**2544[c]**	**139**
Sb	12	0.0039	0.0033	0.0044	0.0030	<0.7	-	<0.5	-
Se	9	0.0230	0.0147	0.0145	0.0070	<4	-	<4	-
Si	15	44	21	51	25	**<15[c]**	**-**	**139[c]**	**36**
Sn	12	0.0039	0.0019	0.0091	0.0053	<0.1	-	0.15	0.11
Sr	18	1.83	0.50	2.35	0.79	1.70	0.43	1.96	0.27
Te	9	0.00093	0.00082	0.00105	0.00056	<1	-	<0.6	-
Ti	18	**0.202[c]**	**0.030**	0.46	0.15	0.74	0.11	**2.82[c]**	**0.40**
Tl	12	0.0027	0.0020	0.0034	0.0019	<4	-	<3	-
U	12	**0.00051[c]**	**0.00012**	**0.00125[c]**	**0.00027**	<3	-	<3	-
V	12	**0.0094[c]**	**0.0052**	**0.0332[c]**	**0.0082**	<0.1	-	<0.1	-
W	3	0.00049	0.00059	0.00037	0.00059	<1	-	<1	-
Zn	18	35	11	28.1	2.0	38.2	5.5	38.3	0.8
Zr	13	0.0099	0.0060	0.0111	0.0032	<0.3	-	<0.1	-

[a] N, number of valid data. [b] M, mean; SD, standard deviation. Digests obtained by methods A or B were analyzed with ICP-MS, and digests obtained by methods B1 or B2 were analysed with ICP-OES. [c,d] Results marked in bold were considered statistically significant for p-values <0.05. Same superscript letters within rows are significantly different. [e] S was determined with ICP-OES.

Table S5. Element concentrations (mg kg^{-1}) in working propolis samples applying four digestion methods prior to analysis by ICP-MS and ICP-OES.

Element	N[a]	Measured concentration[b]							
		Method A		Method B		Method B1		Method B2	
		M	SD	M	SD	M	SD	M	SD
**Al**	19	**152**[c,d]	**29**	260	44	**925**[c]	**86**	**1161**[d]	**52**
**As**	13	0.69	0.19	0.62	0.15	<2	-	<2	-
**B**	19	9.4	1.9	**8.34**[c]	**0.78**	28.4	4.2	**40.1**[c]	**1.6**
**Ba**	19	**1.12**[c]	**0.28**	2.01	0.56	26.7	2.5	**30.0**[c]	**2.0**
**Be**	19	**0.0439**[c]	**0.0068**	0.053	0.013	0.0652	0.0070	**0.082**[c]	**0.012**
**Bi**	13	0.0149	0.0054	0.0192	0.0036	<0.4	-	<0.1	-
**Ca**	19	**1684**[c]	**433**	**1523**[d]	**477**	2105	202	**2602**[c,d]	**122**
**Cd**	19	**0.066**[c]	**0.014**	0.070	0.028	0.180	0.039	**0.234**[c]	**0.020**
**Ce**	19	**1.18**[c]	**0.19**	**1.15**[d]	**0.19**	**0.303**[c,d]	**0.045**	0.384	0.017
**Co**	19	**0.89**[c]	**0.14**	1.08	0.35	1.07	0.11	**1.79**[c]	**0.37**
**Cr**	19	**1.78**[c]	**0.76**	2.07	0.62	4.3	2.0	**4.30**[c]	**0.51**
**Cs**	13	0.283	0.047	0.290	0.039	-	-	-	-
**Cu**	19	10.6	2.7	11.8	7.0	11.1	1.7	18	10
**Fe**	19	1315	392	1624	545	1728	355	2158	496
**Ga**	13	6.6	1.5	10.7	6.9	<1	-	<0.4	-
**K**	19	1682	285	1620	192	1566	113	1927	84
**La**	19	0.60	0.11	0.581	0.094	0.623	0.061	0.786	0.070
**Li**	19	**0.264**[c,d]	**0.056**	0.482	0.073	**0.610**[c]	**0.070**	**0.91**[d]	**0.15**
**Mg**	19	**313**[c]	**43**	377	50	371	17	**442**[c]	**13**
**Mn**	19	**22.4**[c]	**4.9**	23.4	5.2	32.4	2.8	**50.2**[c]	**9.7**
**Mo**	19	0.43	0.12	0.34	0.15	0.280	0.080	0.365	0.089
**Na**	19	273	49	311	99	290	17	419	12
**Nb**	14	0.075	0.016	0.087	0.016	<0.1	-	<0.3	-
**Ni**	19	1.40	0.33	2.07	0.53	1.98	0.55	2.05	0.22
**P**	19	491	152	370	76	503	62	548	9
**Pb**	19	15.3	7.8	15.7	5.4	20.5	6.9	26.2	4.5
**Rb**	13	7.9	1.7	7.13	0.86	<0.6	-	<2	-
**S**[e]	10	271	20	257	35	789	100	870	24
**Sb**	14	0.214	0.041	0.196	0.060	<0.7	-	<0.5	-
**Se**	13	**0.069**[c]	**0.023**	**0.115**[c]	**0.035**	<4	-	<4	-
**Si**	19	**120**[c]	**40**	680	321	1539	537	**4999**[c]	**545**
**Sn**	19	**0.50**[c,d]	**0.11**	1.12	0.35	**1.50**[c]	**0.41**	**1.49**[d]	**0.12**
**Sr**	19	18.7	6.8	19.3	9.2	19.5	4.3	24.6	0.4
**Te**	13	0.0016	0.0014	0.0014	0.0010	<1	-	<0.6	-
**Ti**	19	**7.3**[c,d]	**1.9**	17.6	3.2	**49**[c]	**16**	**203**[d]	**15**
**Tl**	13	0.027	0.011	0.0326	0.0073	<4	-	<3	-
**U**	13	0.041	0.018	0.048	0.011	<3	-	<3	-
**V**	19	**0.69**[c,d]	**0.13**	1.12	0.11	**2.08**[c]	**0.62**	**2.84**[d]	**0.43**
**W**	13	0.046	0.020	0.0263	0.0051	<1	-	<1	-
**Zn**	19	309	80	**258**[c]	**94**	393	121	**487**[c]	**65**
**Zr**	19	**0.57**[c,d]	**0.13**	1.15	0.25	**1.95**[c]	**0.14**	**3.47**[d]	**0.40**

[a] N, number of valid data. [b] M, mean; SD, standard deviation. Digests obtained by methods A or B were analyzed with ICP-MS, and digests obtained by methods B1 or B2 were analysed with ICP-OES. [c,d] Results marked in bold were considered statistically significant for p-values <0.05. Same superscript letters within rows are significantly different. [e] S was determined with ICP-OES.

Table S6. Element concentrations (mg kg^{-1}) in working royal jelly samples applying four digestion methods prior to analysis by ICP-MS and ICP-OES.

Element	N[a]	Measured concentration[b]							
		Method A		Method B		Method B1		Method B2	
		M	SD	M	SD	M	SD	M	SD
**Al**	12	0.337	0.051	0.59	0.21	0.62	0.30	0.86	0.38
**As**	7	0.0073	0.0051	<0.001		<2	-	<2	-
**B**	9	0.607	0.043	0.78	0.17	<13	-	<8	-
**Ba**	9	0.45	0.23	<0.2		0.749	0.082	1.16	0.11
**Be**	8	0.000065	0.000033	0.000081	0.000017	<0.02	-	<0.02	-
**Bi**	6	0.00016	0.00030	0.00039	0.00043	<0.4	-	0.28	0.24
**Ca**	14	309	25	293	30	213	68	176	12
**Cd**	6	0.00066	0.00045	0.00030	0.00028	<0.06	-	<0.02	-
**Ce**	9	0.0023	0.0014	0.00304	0.00071	<0.1	-	<0.4	-
**Co**	7	0.0025	0.0017	0.0025	0.0016	<0.09	-	<0.1	-
**Cr**	12	**0.0120[c]**	**0.0062**	**0.103[c]**	**0.006**	<0.1	-	<0.1	-
**Cs**	8	0.015	0.012	0.0244	0.0035	-	-	-	-
**Cu**	12	5.4	1.0	5.7	1.4	5.92	0.40	6.39	0.10
**Fe**	12	9.1	2.2	10.9	4.2	12.5	1.2	13.3	0.2
**Ga**	5	0.0105	0.0044	<0.00001		<1	-	<0.4	-
**K**	14	3620	600	3310	580	2900	200	3110	72
**La**	8	0.00113	0.00080	0.00142	0.00049	<0.07	-	<0.05	-
**Li**	8	0.0042	0.0021	0.00339	0.00072	<0.1	-	<0.1	-
**Mg**	14	324	33	361	23	323	25	338	9
**Mn**	14	0.80	0.15	0.693	0.061	0.862	0.072	0.913	0.011
**Mo**	8	0.0499	0.0057	0.0614	0.0098	<0.09	-	<0.1	-
**Na**	14	**116[c]**	**23**	132	31	173	13	**183[c]**	**4**
**Nb**	7	0.00015	0.00015	0.00010	0.00017	0.098	0.084	<0.3	-
**Ni**	5	0.016	0.024	0.0297	0.0045	<0.4	-	<0.6	-
**P**	14	**2180[c]**	**200**	2240	303	2800	240	**3010[c]**	**21**
**Pb**	6	0.0052	0.0048	0.0099	0.0030	<0.2	-	<0.2	-
**Rb**	8	17.5	3.0	16.8	3.3	<0.6	-	<2	-
**S[d]**	10	474	40	462	34	1400	160	2050	57
**Sb**	7	0.00093	0.00056	0.00062	0.00019	<0.7	-	<0.5	-
**Se**	8	0.0410	0.0079	0.0363	0.0038	<4	-	<4	-
**Si**	8	**45.3[c]**	**3.5**	**76.6[c]**	**7.8**	<15	-	<15	-
**Sn**	8	0.00221	0.00039	0.00198	0.00084	<0.1	-	<0.1	-
**Sr**	8	0.232	0.047	0.265	0.082	<0.1	-	<0.3	-
**Te**	5	0.00048	0.00039	0.00058	0.00059	<1	-	<0.6	-
**Ti**	11	0.077	0.012	0.102	0.015	<0.05	-	0.108	0.025
**Tl**	8	0.00209	0.00056	0.00214	0.00073	<4	-	<3	-
**U**	8	0.000199	0.000052	0.000272	0.000031	<3	-	<3	-
**V**	5	0.0039	0.0026	0.0013	0.0017	<0.1	-	<0.1	-
**W**	2	0.00044	0.00053	<0.0002		<1	-	<1	-
**Zn**	14	25.4	1.0	25.5	1.9	27.6	3.3	28.9	0.6
**Zr**	8	0.0033	0.0029	0.0032	0.0011	<0.3	-	<0.1	-

[a] N, number of valid data. [b] M, mean; SD, standard deviation. Digests obtained by methods A or B were analyzed with ICP-MS, and digests obtained by methods B1 or B2 were analysed with ICP-OES. [c] Results marked in bold were considered statistically significant for p-values <0.05. Same superscript letters within rows are significantly different. [d] S was determined with ICP-OES.

Table S7. Wavelengths (λ and λ*, nm), linearity range (LLOQ–ULOQ; mg kg^{-1}), and limits of determination (LODs; mg kg^{-1}) for each element and sample digestion treatment (methods A, B, B1, and B2) by ICP-OES.

Element	λ	λ*	LOD_A	LOD_B	LOD_{B1}	LOD_{B2}	N	LLOQ–ULOQ
**Al**	237.312	396.152	1	1	0.6	0.7	4	2 – 50
**As**	188.98	193.696	2	2	2	2	3	5 – 50
**B**	208.889	249.772	5	2	13	8	5	27.5 – 550
**Ba**	233.527	455.403	0.1	0.1	0.2	0.3	3	5 – 50
**Be**	234.861	313.042	0.01	0.01	0.02	0.02	5	1 – 50
**Bi**	190.171	223.061	0.1	0.1	0.4	0.1	4	2 – 50
**C**	193.027	247.856	194	465	300	500	4	1000 – 20000
**Ca**	315.887	317.939	30	30	140	80	5	500 – 10000
**Cd**	214.439	226.502	0.07	0.07	0.06	0.02	4	2 – 50
**Ce**	407.347	418.659	0.3	0.6	0.1	0.4	5	3 – 50
**Co**	228.615	238.892	0.1	0.1	0.09	0.1	4	5 – 50
**Cr**	267.716	284.984	0.2	0.3	0.1	0.1	4	2 – 50
**Cs**	459.311	697.327	-	-	-	-	4	2 – 50
**Cu**	324.754	327.395	0.05	0.05	0.4	0.3	4	2 – 50
**Fe**	238.204	259.94	0.1	0.5	0.3	0.2	3	20 – 100
**Ga**	294.363	417.204	0.7	0.6	1	0.4	4	2 – 50
**K**	766.491	769.897	50	40	140	100	5	250 – 5000
**La**	333.749	379.477	0.07	0.07	0.07	0.05	5	1 – 50
**Li**	460.289	670.783	0.1	0.1	0.1	0.1	3	2 – 10
**Mg**	279.078	280.270	2	1	3	2	5	250 – 5000
**Mn**	257.61	260.568	0.02	0.02	0.05	0.06	4	2 – 50
**Mo**	202.032	204.598	0.05	0.07	0.09	0.1	5	1 – 50
**Na**	589.592	568.821	5	5	10	12	5	250 – 5000
**Nb**	295.088	-	0.1	0.4	0.1	0.3	4	2 – 50
**Ni**	222.295	231.604	0.2	0.3	0.4	0.6	4	2 – 50
**P**	185.878	213.618	20	20	20	20	5	100 – 5000
**Pb**	182.143	220.353	0.1	0.2	0.2	0.2	4	2 – 50
**Rb**	420.179	780.026	0.8	0.2	0.6	2.0	3	5 – 50
**S**	180.669	181.972	20	20	50	20	6	200 – 10000
**Sb**	206.834	217.582	0.4	0.1	0.7	0.5	4	2 – 50
**Se**	196.026	206.279	6	6	4	4	3	10 – 50
**Si**	251.611	288.158	10	10	15	15	4	250 – 5000
**Sn**	189.927	242.95	0.1	0.1	0.1	0.1	4	2 – 50
**Sr**	407.771	460.733	0.1	0.1	0.1	0.3	5	27.5 – 55
**Te**	214.282	238.579	0.3	0.6	1	0.6	4	2 – 50
**Ti**	334.941	336.122	0.2	0.2	0.05	0.1	5	2 – 1010
**Tl**	351.923	377.572	3	3	4	3	3	25 – 100
**U**	263.553	385.464	1	1	3	3	3	5 – 50
**V**	268.796	292.401	0.3	0.3	0.1	0.1	5	1 – 50
**W**	209.475	239.708	3	1	1	1	3	5 – 50
**Zn**	206.2	213.857	0.5	1	3	3	6	20 – 5000
**Zr**	339.198	-	0.3	0.3	0.3	0.1	4	2 – 50

Table S8. Element concentrations of the certified material (NIST 1515; n=6; mg kg^{-1}) obtained by ICP-MS (methods A, B, and B1) and ICP-OES (method B2).

Element	NIST 1515[a]	Measured concentration[b]							
		Method A	Z-score[c]	Method B	Z-score[c]	Method B1	Z-score[c]	Method B2	Z-score[c]
**Al**	284.5 ± 5.8	248 ± 31	-2.8	257 ± 10	-6.5	274 ± 12	-2.1	254 ± 15	-5.0
**As**	–	0.118 ± 0.026	–	0.149 ± 0.051	–	<2	–	<2	–
**B**	27.6 ± 2.8	17.7 ± 0.6	-31.1	17.0 ± 0.6	-43	30.0 ± 1.2	2.4	24.5 ± 1.4	-4.3
**Ba**	48.8 ± 2.3	44.1 ± 0.7	-13.7	42.5 ± 0.4	-35.5	49.0 ± 5.2	0.1	43.4 ± 2.6	-4.2
**Be**	–	0.00872 ± 0.00020	–	0.0104 ± 0.0002	–	0.0126 ± 0.0012	–	<0.02	–
**Bi**	–	0.00278 ± 0.00060	–	0.00286 ± 0.00028	–	0.00359 ± 0.00013	–	<0.1	–
**Ca**	15250 ± 100	15300 ± 65	0.2	14800 ± 97	-2.1	16400 ± 3000	0.8	12700 ± 670	-7.5
**Cd**	0.0132 ± 0.0015	0.0126 ± 0.0017	-0.7	0.0129 ± 0.0002	-4.3	0.0120 ± 0.0005	-5.4	<0.02	–
**Ce**	3	2.46 ± 0.01	-132	2.80 ± 0.01	-50.0	2.81 ± 0.27	-1.4	2.74 ± 0.70	-0.9
**Co**	0.09	0.0978 ± 0.0014	46.2	0.101 ± 0.002	31.6	0.107 ± 0.008	4.1	<0.1	–
**Cr**	0.3	0.080 ± 0.018	-24.6	0.305 ± 0.056	0.2	0.327 ± 0.056	1.2	0.327 ± 0.008	6.5
**Cs[d]**	–	0.00391 ± 0.00030	–	0.00525 ± 0.00013	–	0.00600 ± 0.00041	–	–	–
**Cu**	5.69 ± 0.13	4.59 ± 0.13	-21.4	5.44 ± 0.16	-3.8	5.58 ± 0.26	-1.0	5.43 ± 0.25	-2.1
**Fe**	82.7 ± 2.6	79.5 ± 9.3	-0.7	88.1 ± 2.0	4.9	89.2 ± 6.2	2.1	65.9 ± 3.1	-10.7
**Ga**	–	0.0735 ± 0.0023	–	0.115 ± 0.008	–	0.304 ± 0.021	–	<0.4	–
**K**	16080 ± 210	14000 ± 184	-22.5	15300 ± 393	-4.2	16000 ± 1200	-0.2	13600 ± 55	-91.8
**La**	20	16.4 ± 0.3	-24.8	17.8 ± 0.3	-15.0	18.1 ± 1.5	-1.7	18.0 ± 2.6	-1.5
**Li**	–	0.0226 ± 0.0015	–	0.0856 ± 0.0003	–	0.0886 ± 0.0050	–	0.100 ± 0.001	–
**Mg**	2710 ± 120	2560 ± 12	-31	2590 ± 22	-10.7	2740 ± 150	0.4	2490 ± 69	-6.5
**Mn**	54.1 ± 1.1	59.3 ± 4.7	2.2	55.5 ± 2.5	1.1	55.8 ± 3.3	1.1	47.1 ± 2.1	-6.7
**Mo**	0.095 ± 0.011	0.0793 ± 0.0029	-10.8	0.0820 ± 0.0046	-5.6	0.092 ± 0.013	-0.6	<0.1	–
**Na**	24.4 ± 2.1	27.9 ± 0.2	38.3	28.4 ± 1.1	7.7	27.1 ± 3.4	1.6	27.1 ± 0.9	6.0
**Nb**	–	0.00220 ± 0.00018	–	0.0116 ± 0.0002	–	0.0285 ± 0.0016	–	<0.3	–
**Ni**	0.936 ± 0.094	0.982 ± 0.022	4.2	0.938 ± 0.012	0.4	1.04 ± 0.12	2.2	0.811 ± 0.031	-8.0
**P**	1593 ± 68	1640 ± 11	-3.3	1570 ± 25	-0.7	1580 ± 36	-1.5	1620 ± 84	0.5
**Pb**	0.470 ± 0.024	0.372 ± 0.024	-8.2	0.475 ± 0.040	0.2	0.444 ± 0.027	-1.9	0.59 ± 0.10	2.3
**Rb**	10.2 ± 1.6	9.37 ± 0.42	-4.4	9.34 ± 0.46	-4.2	9.33 ± 0.43	-4.5	6.54 ± 0.39	-18.8
**S[e]**	1800	1862 ± 56	2.9	1870 ± 52	3.1	1930 ± 51	6.2	2000 ± 250	1.6
**Sb**	0.013	0.0120 ± 0.0028	-0.9	0.0128 ± 0.0011	-0.4	0.0119 ± 0.0029	-0.9	<0.5	–
**Se**	–	0.055 ± 0.010	–	0.051 ± 0.023	–	<4	–	<4	–

**Si**	–	432 ± 22	–	573 ± 22	–	550 ± 30	–	647 ± 65	–
**Sn**	–	0.0359 ± 0.0015	–	0.0451 ± 0.0017	–	0.0497 ± 0.0002	–	0.27 ± 0.24	–
**Sr**	25.1 ± 1.1	25.4 ± 1.2	0.6	23.7 ± 0.2	-11.6	22.9 ± 3.5	-1.2	21.2 ± 0.6	-13.8
**Te**	–	0.0033 ± 0.0010	–	0.0034 ± 0.0034	–	0.0094 ± 0.0020	–	<0.6	–
**Ti**	–	0.62 ± 0.13	–	3.9 ± 1.4	–	11.5 ± 1.6	–	12.5 ± 0.8	
**Tl**	–	0.00885 ± 0.00076	–	0.00803 ± 0.00023	–	0.0097 ± 0.0021	–	<3	–
**U**	0.006	0.00325 ± 0.00020	-28.0	0.00415 ± 0.00016	-22.6	0.00548 ± 0.00056	-2.2	<3	–
**V**	0.254 ± 0.027	0.239 ± 0.015	-2.0	0.274 ± 0.032	1.3	0.250 ± 0.014	-0.6	0.235 ± 0.039	-1.0
**W**	0.007	0.0085 ± 0.0037	0.8	0.00479 ± 0.00013	-34.1	0.0074 ± 0.0013	0.8	<1	–
**Zn**	12.45 ± 0.43	11.2 ± 0.9	-3.1	11.8 ± 0.4	-3.8	12.4 ± 0.2	-0.6	11.1 ± 0.7	-4.9
**Zr**	–	0.0323 ± 0.0004	–	0.0586 ± 0.0021	–	0.0689 ± 0.0033	–	0.29 ± 0.21	–

[a] Information mass fraction values for elements in SRM 1515. [b] ±Uncertainty = (k × Xf × CVr)/100 × $\sqrt{n}$; Xf = mean of observed values, CVr = repeatability as coefficient of variation percent intra-run (n = 6), and k = 2 (p = 95%). [c] Z-score = (Xf − Xc)/(SDr/$\sqrt{n}$) where Xc is the certified value and SDr the standard deviation for the repeatability precision. [d] Cs could not be analyzed because axial view ICP-OES is strongly affected by ionisation interference when elements with low ionisation potentials coexist in the sample. [e] S concentration is determined with ICP-OES.

Table S9. Trueness bias percentage (Tbias%) and repeatability (CVr%) of the certified material (NIST 1515; n=6; mg kg^{-1}) obtained by ICP-MS (methods A, B, and B1) and ICP-OES (method B2).

	Method A		Method B		Method B1		Method B2	
**Element**	**Tbias%**	**CVr%**	**Tbias%**	**CVr%**	**Tbias%**	**CVr%**	**Tbias%**	**CVr%**
**Al**	-12.8	12.7	-9.7	4.0	-3.7	4.5	-10.7	5.8
**B**	-35.9	4.4	-38.3	3.5	8.7	8.0	-11.1	7.1
**Ba**	-9.6	1.9	-12.9	1.0	0.4	13.0	-11.1	7.3
**Ca**	0.3	3.0	-2.9	3.4	7.6	22.2	-16.6	6.5
**Cd**	-4.6	16.2	-2.6	1.6	-8.9	4.4	nd[a]	nd[a]
**Ce**	-18.0	0.4	-6.8	0.4	-6.4	11.6	-8.7	25.5
**Co**	8.7	1.4	12.2	1.8	12.6	11.2	nd[a]	nd[a]
**Cr**	-73.4	27.6	1.6	22.6	9.0	17.0	9.1	3.2
**Cu**	-19.4	2.8	-4.3	2.9	-1.9	4.6	-4.6	5.6
**Fe**	-3.9	14.3	6.5	3.0	7.9	8.5	-20.3	5.8
**K**	-12.9	1.6	-5.1	3.2	-0.7	9.2	-15.7	0.5
**La**	-17.8	2.1	-10.9	2.0	-9.7	15.5	-9.9	17.6
**Mg**	-5.6	0.5	-4.3	1.0	1.0	6.7	-8.2	3.4
**Mn**	9.6	9.8	2.7	5.5	3.2	7.2	-12.9	5.4
**Mo**	-16.5	4.5	-13.7	6.9	-3.6	14.5	nd[a]	nd[a]
**Na**	14.3	0.8	16.6	4.5	11.0	15.3	11.0	4.1
**Ni**	4.9	2.7	0.2	1.5	11.3	11.2	-13.4	4.7
**P**	3.1	0.9	-1.6	2.6	-1.0	3.5	1.4	6.4
**Pb**	-20.9	7.9	1.1	10.4	-5.5	7.4	24.8	20.8
**Rb**	-8.1	4.9	-8.4	5.4	-8.5	5.0	-35.9	7.3
**S[b]**	3.4	2.8	3.9	3.0	7.2	2.6	11.4	15.3
**Sb**	-7.7	23.6	-1.4	9.0	-8.5	24.6	nd[a]	nd[a]
**Sr**	1.3	5.6	-5.5	1.2	-8.6	18.6	-15.7	3.3
**U**	-45.8	7.4	-30.9	4.8	-8.6	10.3	nd[a]	nd[a]
**V**	-5.9	7.6	7.8	14.1	-1.6	7.1	-7.5	20.4
**W**	22.0	52.5	-31.6	3.3	5.9	18.1	nd[a]	nd[a]
**Zn**	-10.0	8.8	-5.2	3.6	-0.7	3.2	-10.5	5.9

[a] nd, not determined because the element concentration was lower than LOD. [b] S concentration is determined with ICP-OES.

Table S10. Concentrations (mg Kg^{-1}) of each element in some commercially beehive products obtained by method B.

Element	Honey 1		Honey 2		Honey 3		Honey 4		Honey 5	
	M	SD	M	SD	M	SD	M	SD	M	SD
**Al**	0.61	0.12	0.318	0.063	7.74	0.49	5.60	0.22	0.597	0.071
**As**	<LOD	-	<LOD	-	<LOD		<LOD		<LOD	-
**B**	6.23	0.30	0.671	0.027	3.28	0.12	3.63	0.28	7.22	0.35
**Ba**	<LOD	-	<LOD	-	1.722	0.025	0.503	0.045	<LOD	-
**Be**	<LOD	-	<LOD	-	0.00116	0.00023	0.00139	0.00023	<LOD	-
**Bi**	<LOD	-	<LOD	-	0.000261	0.000053	<LOD	-	<LOD	-
**Ca**	83.0	8.2	32.5	8.1	147	10	129	17	55	14
**Cd**	0.000177	0.000028	0.00067	0.00023	0.00159	0.00047	<LOD	-	<LOD	-
**Ce**	0.00104	0.00023	<LOD	-	0.0131	0.0010	0.0184	0.0004	0.000968	0.000059
**Co**	0.00597	0.00057	0.00144	0.00037	0.0105	0.0005	0.0105	0.0010	0.00151	0.00036
**Cr**	0.0129	0.0016	0.0377	0.0099	0.0204	0.0029	0.0153	0.0040	<LOD	-
**Cs**	0.00412	0.00020	0.00049	0.00010	0.696	0.012	0.502	0.032	0.00250	0.00039
**Cu**	0.301	0.038	0.076	0.017	0.764	0.034	1.02	0.10	0.150	0.020
**Fe**	3.10	0.19	2.20	0.56	3.98	0.94	4.59	0.57	0.742	0.081
**Ga**	0.00248	0.00038	<LOD	-	0.0378	0.0043	0.0124	0.0029	<LOD	-
**K**	1330	20	96.7	6.0	4700	204	3791	223	750	28
**La**	0.00050	0.00010	0.000172	0.000038	0.00800	0.00054	0.00991	0.00023	0.00070	0.00016
**Li**	0.00938	0.00089	0.00918	0.00051	0.00334	0.00028	0.0107	0.0004	0.00442	0.00020
**Mg**	47.5	3.1	21.1	0.9	91.1	3.1	107	3	18.9	0.9
**Mn**	1.07	0.05	0.314	0.024	2.13	0.23	2.09	0.17	0.120	0.022
**Mo**	0.00444	0.00093	0.00054	0.00018	0.0080	0.0011	0.0056	0.0010	0.00166	0.00032
**Na**	51.5	1.7	109	6	32.7	2.8	51.7	0.9	16.8	2.1
**Nb**	0.0000203	0.0000028	<LOD	-	0.00093	0.00025	0.00100	0.00029	0.000053	0.000010
**Ni**	0.0357	0.0059	0.0217	0.0036	0.0391	0.0021	0.0586	0.0092	0.0090	0.0021
**P**	54.9	7.8	10.6	0.8	65.5	2.4	136	9	32.4	1.7
**Pb**	0.0286	0.0059	0.00199	0.00055	0.0296	0.0075	0.0347	0.0049	0.0234	0.0081
**Rb**	1.76	0.11	0.0983	0.0076	59	12	49.2	8.9	1.54	0.18
**Sb**	<LOD	-	0.00201	0.00071	<LOD	-	<LOD	-	0.00208	0.00083
**Se**	<LOD	-	<LOD	-	<LOD	-	<LOD	-	<LOD	-
**Si**	43.0	8.9	32.4	3.9	27.3	0.9	48.5	4.5	51.9	2.2
**Sn**	0.0055	0.0019	0.00108	0.00016	0.0137	0.0019	0.00494	0.00043	0.0028	0.0008
**Sr**	0.494	0.004	0.102	0.020	1.15	0.10	1.14	0.22	0.175	0.032
**Te**	<LOD	-	<LOD	-	0.0056	0.0015	0.0053	0.0013	0.0023	0.0006
**Ti**	0.0633	0.0028	0.0408	0.0072	0.160	0.012	0.183	0.025	0.056	0.012
**Tl**	0.000176	0.000011	0.000205	0.000054	0.0378	0.0016	0.298	0.024	0.000387	0.000049
**U**	0.000105	0.000013	0.000182	0.000043	0.00117	0.00020	0.00143	0.00010	0.000093	0.000032
**V**	<LOD	-	<LOD	-	0.0170	0.0033	0.0207	0.0062	0.0071	0.0024
**W**	<LOD	-	<LOD	-	<LOD	-	<LOD	-	<LOD	-
**Zn**	1.62	0.07	<LOD	-	0.82	0.16	2.14	0.26	<LOD	-
**Zr**	0.00128	0.00028	<LOD	-	0.0141	0.0011	0.0190	0.0040	0.00058	0.00014

Table S11. Concentrations (mg Kg^{-1}) of each element in some commercially beehive products obtained by method B.

Element	Honeydew 1		Honeydew 2		Beeswax		Pollen		Royal jelly	
	M	SD	M	SD	M	SD	M	SD	M	SD
**Al**	1.39	0.13	4.43	0.19	0.38	0.15	6.76	0.36	0.624	0.032
**As**	<LOD	-	<LOD		0.0385	0.0061	0.013	0.022	<LOD	-
**B**	4.09	0.26	7.17	0.26	<LOD	-	4.96	0.45	1.45	0.16
**Ba**	0.060	0.015	0.9	0.1	<LOD	-	1.29	0.10	<LOD	-
**Be**	<LOD	-	0.00244	0.00023	<LOD	-	0.00051	0.00018	<LOD	-
**Bi**	0.000400	0.000072	0.000301	0.000082	<LOD	-	<LOD	-	<LOD	-
**Ca**	20.2	4.5	115	9	86	19	441	97	159	41
**Cd**	0.00120	0.00024	0.00069	0.00018	<LOD	-	0.085	0.014	<LOD	-
**Ce**	0.00279	0.00067	0.0404	0.0020	0.00100	0.00023	0.0086	0.0016	0.00054	0.00013
**Co**	0.0167	0.0010	0.0261	0.0016	<LOD	-	0.177	0.020	0.00195	0.00030
**Cr**	0.0282	0.0048	0.0682	0.0091	0.0308	0.0018	0.24	0.13	0.0370	0.0040
**Cs**	0.00339	0.00016	0.521	0.026	0.00039	0.00020	0.0114	0.0022	0.00079	0.00017
**Cu**	1.95	0.08	3.51	0.19	<LOD	-	6.16	0.36	4.75	0.16
**Fe**	2.91	0.21	5.20	0.38	0.324	0.040	33	4	10.7	0.7
**Ga**	0.00169	0.00043	0.0179	0.0010	<LOD	-	0.0328	0.0079	<LOD	-
**K**	6540	270	2550	131	<LOD	-	4237	312	2750	120
**La**	0.00142	0.00026	0.0165	0.0011	<LOD	-	0.0043	0.0011	0.000383	0.000077
**Li**	0.00933	0.00037	0.00512	0.00037	<LOD	-	0.0169	0.0011	0.00378	0.00050
**Mg**	105	5	126	6	2.8	2.2	381	25	262	9
**Mn**	1.17	0.04	2.69	0.14	0.039	0.016	19	0	0.701	0.054
**Mo**	0.0844	0.0044	0.00363	0.00082	<LOD	-	0.044	0.013	0.0502	0.0018
**Na**	10.0	1.2	44.3	1.0	148	9	24.2	4.5	103	5
**Nb**	0.000151	0.000036	0.00119	0.00008	<LOD	-	<LOD	-	0.000032	0.000029
**Ni**	1.04	0.06	0.0342	0.0043	<LOD	-	1.71	0.01	<LOD	
**P**	225	11	180	9	<LOD	-	1636	294	1020	26
**Pb**	0.0208	0.0040	0.0211	0.0026	0.0124	0.0066	0.0203	0.0048	0.0067	0.0014
**Rb**	3.54	0.12	23.1	2.3	0.0179	0.0032	3.8	1.8	2.17	0.07
**Sb**	0.0080	0.0025	0.000894	0.000082	0.0046	0.0024	<LOD	-	0.00037	0.00029
**Se**	<LOD		<LOD	-	<LOD	-	<LOD	-	0.025	0.013
**Si**	71.2	7.9	64.2	1.0	214	39	50.9	9.5	50.3	4.5
**Sn**	0.0103	0.0018	0.00490	0.00041	0.0059	0.0011	0.00572	0.00020	0.338	0.022
**Sr**	0.0713	0.0043	2.71	0.29	<LOD	-	0.840	0.036	0.0373	0.0051
**Te**	<LOD		<LOD		<LOD	-	0.0025	0.0020	0.00101	0.00008
**Ti**	0.0910	0.0009	0.262	0.043	0.040	0.018	0.93	0.25	0.205	0.014
**Tl**	0.000325	0.000010	0.0114	0.0008	<LOD	-	<LOD	-	0.000809	0.000055
**U**	0.000249	0.000021	0.00144	0.00012	0.000203	0.000055	0.000306	0.000072	0.000163	0.000034
**V**	<LOD		0.0363	0.0059	0.168	0.023	0.71	0.33	0.0059	0.0015
**W**	<LOD		<LOD		<LOD	-	<LOD	-	<LOD	-
**Zn**	0.41	0.11	2.56	0.13	2.40	0.22	24.1	3.3	22.5	0.7
**Zr**	0.00059	0.00010	0.0293	0.0041	0.00118	0.00027	0.00592	0.00055	0.00017	0.00011

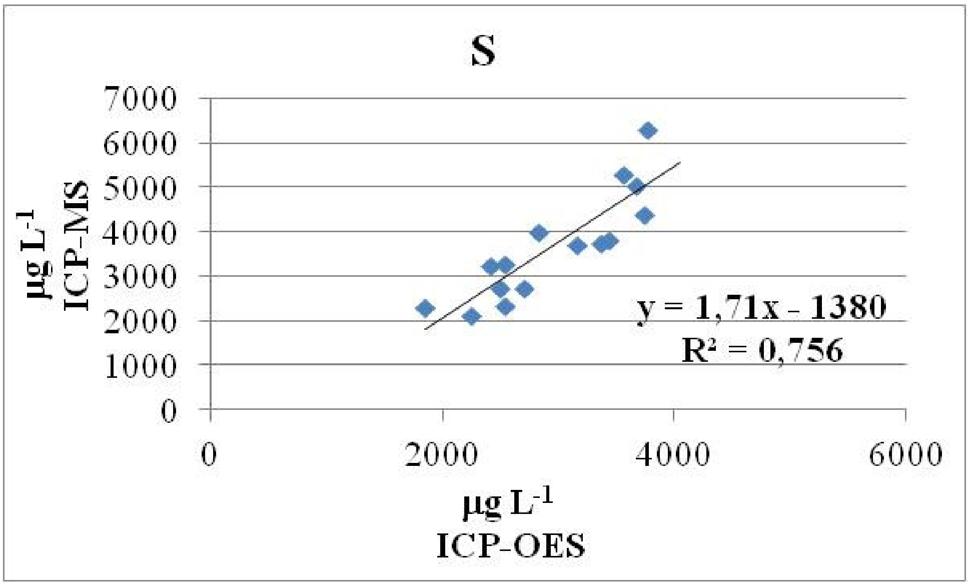

Figure S1. Correlation between the S data obtained in working samples by ICP-MS and ICP-OES.
